# What are health professionals’ intentions toward using research and products of research in clinical practice? A systematic review and narrative synthesis

**DOI:** 10.1002/nop2.40

**Published:** 2015-12-17

**Authors:** Ben Appleby, Carolyn Roskell, William Daly

**Affiliations:** ^1^ Faculty of HealthEducation and Life SciencesSchool of NursingMidwifery and Social WorkBirmingham City UniversityEdgbastonBirminghamUK; ^2^ College of Life and Environmental SciencesSchool of Sport, Exercise and Rehabilitation SciencesUniversity of BirminghamBirminghamUK; ^3^ College of Medical and Dental SciencesSchool of Health and Population SciencesNursing, University of BirminghamBirminghamUK

**Keywords:** Attitude, behaviour, clinical context, clinical guidelines, health professionals, intention, nurses, nursing, practice habit, research utilization, social cognitive models of behaviour

## Abstract

**Aim:**

To explore health professionals’ intentional behaviour and what determines their intention to use products of research in clinical practice.

**Background:**

Trying to get research and products of research into clinical practice is an enduring problem. A clearer picture is emerging as to how individual practitioners respond toward practical problems of changing clinical practice, but this does not include health professionals’ intentions to use products of research and what influences their intentions.

**Design:**

Systematic Review and Narrative Synthesis.

**Data sources:**

Five databases were searched systematically. This included BNI, HMIC, Psych INFO, CINHAL and MEDLINE; articles published in the English language only were included.

**Review methods:**

PRISMA guidelines were used as a framework for structuring the review and methods of narrative synthesis to analyse study outcomes.

**Results:**

Eighteen studies matched the final inclusion criteria. All studies used questionnaires to measure intention. Most studies involved Nurses or Physicians. Nurses’ intentions were mostly influenced by their perceived ability to use guidelines in their practice. Physicians’ intentions were often influenced by their perceptions of the usefulness and relevance of the guideline and peer pressure amongst the professional group. Practice habits, when added to intentional models were also predictive of intentional behaviour. In studies that compared intentions with behaviour, the level of intention often did not match self‐report or actual behaviour.

## Introduction

The problem of getting healthcare professionals to use research and products of research in their practice appears to be an ongoing, almost intractable problem. Over the last fifteen years numerous reports have highlighted the continuing gap between what is known to be best practice and the care that patients receive (McGlynn *et al*. 2003, Hussey *et al*. 2004, Madon *et al*. 2007). This continuing gap has been recognized as an international problem in developed and underdeveloped countries (Straus *et al*. 2009). Therefore, despite producing increasing quantities of high quality evidence it would appear that the slow uptake or failure to adopt evidence still persists.

Traditionally, understanding the reasons *why* research and products of research were not implemented in practice focused on identifying multiple individual and organizational barriers, mostly using survey methods (Cabana *et al*. 1999, Hutchinson and Johnstone 2006, Bostrom *et al*. 2008, Kajermo *et al*. 2010) and the barriers scale to research implementation (Funk *et al*. 1991a). The use of this type of questionnaire to identify health professionals’ barriers and facilitators to using research continues to be used (Salbach *et al*. 2010, Cahill *et al*. 2013, Straus *et al*. 2013, Weng *et al*. 2013, Zardo & Collie 2014) and does help to identify differences in knowledge, skills, attitudes and beliefs and behaviours. However, evidence has suggested that using multidimensional scales in questionnaire surveys to target practitioner behaviour change is often ineffective (Davis *et al*. 1995, Watson & Myres 2001, Jenner *et al*. 2002).

At the same time, exploration of health professionals’ use of research in clinical practice has also focused specifically on their behaviour and individual disposition. Empirical reviews exploring determinates of research use behaviour have identified attitudes and beliefs as the only significant determinants (Estabrooks *et al*. 2003, Squires *et al*. 2011). The identification of attitudes and beliefs highlighted ‘individual disposition’ as an important influence in a health professional's research utilization behaviour and also recognized the need to analyse internal factors or structures influencing behaviour rather than just external factors (Grimshaw *et al*. 2001, 2004). Consequently, a plethora of research using a variety of methods has explored health professionals’ attitudes and beliefs toward using research in different areas of practice (Barnard & Wiles 2001, Bjorkstrom & Hamrin 2001, Bonner & Sando 2008, Munroe *et al*. 2008, Weng *et al*. 2013, Kajermo *et al*. 2014). However, in these studies, the exploration of attitudes and beliefs were not extended to understanding the relationship with intention.

The importance of understanding the role of individual behaviour (and intention) has been explored by the use of theory (Grol 2001) and more recently as part of wide‐ranging theoretical frameworks. The Theoretical Domains Framework (TDF) is a good example and represents an integrated theoretical framework of several domains and theoretical constructs (including intention) synthesized from 33 theories and 128 constructs (Michie *et al*. 2005). The framework has been used to explore dementia (Murphy *et al*. 2014), low back pain (McKenzie *et al*. 2008, 2010), hand hygiene (Boscart *et al*. 2012, Dyson *et al*. 2013) and many more clinical behaviours all helping to explain elements of health practitioner behaviour. The TDF has also been used to evaluate behavioural barriers to specific interventions (Dyson *et al*. 2013), to uncover individual's beliefs (Murphy *et al*. 2014) and individual barriers to using clinical guidelines (Dyson *et al*. 2013). This demonstrates the increasing empirical and clinical interest in understanding individual health professional behaviour. However, the framework has not been used to evaluate intentional research use behaviour.

Theoretically based social cognitive psychological models such as the Theory of Planned Behaviour (TPB) provide a reliable platform for exploring reasoned behaviour and the attitudes and beliefs which influence individual behaviour (Godin *et al*. 2008). The theory assumes that individuals are rational actors who carefully process information before making intentional, volitional decisions (Ajzen 1991). Godin *et al*. (2008) systematic review established that health professionals’ do make intentional choices as part of their professional behaviour and intentional models can capture and predict large proportions of behaviour. Continued application of intentional models in understanding behaviour is important, as this will provide a reliable structure for changing clinical behaviour (Eccles *et al*. 2005). Moreover, it is also more probably that theory‐based information can provide more generalizable solutions to changing behaviour (Murphy *et al*. 2014). Therefore, in this review, primary studies which used intention based models formed the basis for the review of evidence.

Research utilization was used as a construct to help focus the exploration of health professionals’ use of research. The construct is said to be composed of ‘instrumental’ ‘symbolic’ and ‘conceptual’ components (Larsen 1980, Beyer & Trice 1982, Estabrooks 1999). Instrumental utilization generally refers to the actual use of research products in clinical practice to help guide or inform practice, ‘conceptual utilization’ involves professional reasoning when using research and ‘symbolic utilization’ is when research is related to but does not necessarily inform a decision (Estabrooks *et al*. 2003).

In a more recent analysis of the construct, Standberg *et al*. (2013) suggested that when these concepts are applied to practice, the process of behaviour involves either ‘deliberative’ or ‘passive’ processes of thinking. These processes of thought are clearest and most apparent when applied to instrumental research utilization. In Standberg *et al*. (2013) study, nurses’ could clearly identify examples of ‘instrumental research utilization’ but could not distinguish between ‘conceptual’ and ‘symbolic’ definitions. As this evidence suggests, there is difficulty in trying to distinguish between ‘conceptual’ and ‘symbolic’ definitions of research utilization behaviour, which has implications to the measurement of intention and inclusion of evidence in this review.

The measurement of intention is most reliable when the ‘intention’ is clearly aligned to a recognizable ‘behaviour’ (Ajzen 1991). As the description of ‘conceptual’ and ‘symbolic’ research utilization are hard to distinguish, then the measurement of intention is likely to be unstable. Because of the lack of clarity of these concepts it could also be that the empirical measurement of intention is limited. Moreover, as the identification and synthesis of evidence in this review requires the clear identification of research utilization behaviour, omitting ‘conceptual’ and ‘symbolic’ definitions avoided confusion in the application of review processes. On this basis the review proceeded to focus on ‘instrumental’ and not ‘conceptual’ or ‘symbolic’ definitions of research utilization.

It is clearly evident that behavioural decisions are made when nurses use protocols in clinical practice; decisions are not entirely passive. Standberg *et al*. (2013) conceptual analysis has identified that nurses’ instrumental research activity involves a cognitively active or passive process of thought. Examining the active (deliberative) or passive processes involved through intention will enhance our understanding of this behaviour. It is also unclear as to what determines these thinking processes and how or if these might change to different types of instrumental research behaviour. A review of the evidence can help to provide these missing answers.

This review will therefore focus on exploring individual health professionals’ intention and determinates of intention to use products of research (e.g. clinical guidelines, protocols or decision‐aids) directly in their practice. This approach has been taken to evaluate whether evidence related to health professionals’ ‘intention’ to use research products may bring new understanding to how health professionals’ use evidence in their practice.

### The need for a review of the literature

Over the last 15–20 years implementation research has identified important barriers and facilitators to changing practice. Multiple pragmatic dimensions, individual attitudes and beliefs and theoretical frameworks of behaviour have added and provided direction for exploring implementation behaviour. However, there is still a need to explore the empirical evidence to explain health professionals’ intentions when ‘products of research’ are used to guide practice.

Empirically, previous reviews of intentional behaviour have explored general health professional behaviour and not specifically instrumental research utilization behaviour. Perkins *et al*. (2007) systematic review focused on exploring health professionals’ general behaviour in the context of one type of motivational theory – The Theory of Reasoned Action. Similarly, Godin *et al*. (2008) systematic review explored the explanatory value of a range of social cognitive models (e.g. Theory of Reasoned Action, Theory of Planned Behaviour) on general health professionals’ behaviour, although this included very few studies that would fit the definition of instrumental research utilization.

However, no review (of any methodology) has been conducted to explain a health professional's intention to use products of research to guide their practice. Thus, there is a need to systematically scrutinize the available literature using reliable review processes and methods to address this area of research.

### Operational definitions: intention and intentional behaviour

Intention represents an individual's planned and rationalized decision to carry out a behaviour (Ajzen 1991). Intentional decisions are referred to as ‘intentional behaviour’ or just ‘intention’ (Ajzen 1991). In effect these two terms share the same meaning.

In all studies, a form of research utilization behaviour is identified. In some studies only the intention to carry out that behaviour is reported. In other studies, intention and the relationship with behaviour is also measured. In approximately half of the studies, the relationship between intention and behaviour is not analysed because intention is considered to be a reliable proximal measure of behaviour (Eccles *et al*. 2006). In very few studies an individual's ‘actual behaviour’ is also measured for a comparison with intention. This conceptual understanding of intention applies to all models of intention identified in the review.

For clarity, in this review the instrumental research utilization behaviour will be clearly stated. At all times, the term ‘intention’ will be used to refer to an individual's intentional behaviour. If a study has not reported the relationship between intention and behaviour, this will be recorded as missing. Actual observed behaviour (if reported) will be referred to as ‘actual behaviour’.

## The review

### Aim

The overarching aim of this study was to explore health professionals’ intentions to using products of research in clinical practice, in relation to *instrumental* definitions of research utilization behaviour, and address the following review questions:

- Are there professional differences or similarities in intentional instrumental research utilization behaviours?
- Are there other influences on intentional behaviour in addition to those explained by social cognitive model variables?
- In which circumstances is intention a powerful predictor of instrumental research utilization behaviour?
- Is there a consistent pattern of determinates of intentional instrumental research utilization behaviour?

### Methodological objectives

The main methodological objectives are to review empirical evidence using well established review methods, to provide clear answers to the main review aim. Methodological objectives are:

- To develop a systematic search strategy to search for, acquire and select primary empirical evidence relevant to the review questions and aim
- To subject selected literature to a rigorous appraisal of methodological quality using appropriate tools
- To draw conclusions as to the current status and quality of evidence relating to the review question, to make a further contribution to the field.

### Design

The design of the review was driven by the need to follow systematic processes and also to provide a narrative interpretation of collective outcomes across studies. The Preferred, Reporting Items of Systematic Reviews and Meta‐Analysis (PRISMA) statement was used as a guide for the systematic review of studies (Moher *et al*. 2009). Popay *et al*.’ (2006) guide to narrative synthesis was used to integrate and interpret key findings across empirical studies.

### Search methods

The main literature search involved a focused electronic search on key health related databases, these included the BNI (British Nursing Index) 1985–October 2011; HMIC (Health Management Information Consortium made up of 2 databases DH‐data Department of Health's Library and Information Services and King's Fund Information and Library Service) 1983–October 2011; PsycINFO (database of abstracts of psychological literature) 1806–October 2011; CINAHL (Cumulative Index of Nursing and Allied Health Literature) 1981–October 2011; MEDLINE (Medical Literature Analysis and Retrieval System) 1950–October 2011 and the Cochrane Library to October 2011. All databases were accessed via NHS Evidence (www.library.nhs.uk).

### Search strategy

Database searching was developed in conjunction with an information specialist. As not all databases use the same controlled vocabulary, different search terms were used as applicable. Both free‐text and thesaurus terms specific to each database were used to create a maximally sensitive search strategy. Terms were combined using the Boolean AND/OR and where appropriate truncation (*) was applied to retrieve variations on a word stem. The following are examples that represent the population, exposure and outcome: (nurs*): title, abstract, keyword and (evidence or research): ti,ab,kw and (attitude* or inten* or engag* or motivat* or ‘perceived social norm*’ or ‘social behaviour’ or ‘social behavior’ or ‘peer pressure*’ or determinant near/5 behaviour* or determinant near/5behavior*):ti,ab,kw. Both UK and US terminology were used for the search. No date limits were applied, to maximize search by date. Hand searching and referencing chaining for relevant empirical studies (for sake of consistency) stopped when electronic searches were completed.

The initial search strategy was updated and re‐run in May 2015; this identified the final number of primary studies which matched the inclusion criteria. The final search strategy also involved scanning specialist journals in Implementation Science.

### Inclusion criteria

The inclusion criteria for study selection were influenced by the review question and associated aims of the review (Table 1). Primary empirical studies were included if they clearly related to Health Professionals’ intentions to use research products directly in their practice. Only studies which measured intention through theoretical models were included.

**Table 1:** Inclusion criteria for empirical studies

Studies were accepted that met the inclusion criteria according to participants, types of exposure, type of behaviour, study design and language.
*Participants*: Health professionals such as nurses, doctors, physiotherapists, midwives, radiographers, speech and language therapists and health personnel undertaking instrumental research utilization activities such as healthcare support workers or laboratory workers.
*Exposure*: Primary empirical studies that clearly measured intentional instrumental research utilization behaviour delivered in practice.
*Outcome:* Primary empirical studies must measure a recognized activity of instrumental research utilization in relation to clinical practice such as: use of clinical guidelines, protocols or decision‐aids.
*Design:* Primary empirical studies that measured instrumental intentional behaviour. These could include a variety of designs with the predominant design being observational, descriptive analytical studies.
*Language*: Published articles in English

### Exclusion criteria

Systematic Reviews are strengthened by providing a rationale for exclusion criteria. Studies that did not meet the inclusion criteria in Table 2 were subsequently excluded.

**Table 2:** Exclusion criteria of empirical studies

Studies with any of the following elements were excluded from the review.
*Participants*: Studies that did not focus on health professionals or health personnel. Studies were excluded if the focus was on non‐health professionals and students, regardless of student degree programme.
*Exposure*: Empirical studies that did not measure intention. Empirical studies that measured the determinates of instrumental research utilization intention (e.g. attitude, social pressure) but did not relate this to intention were excluded.
*Outcome:* Primary empirical studies must measure a recognized activity of instrumental research utilization behaviour in relation to clinical practice. Empirical studies were not included if intentions were not clearly linked to a research utilization activity.
*Design:* Non‐empirical studies. Any opinion based articles without an empirical method were excluded. Secondary evidence (any type of review) was also excluded as the choice of synthesis precludes the integration of primary and secondary evidence.
*Language*: Published articles, languages other than English

### Search outcome

The screening/filtering process involved 3 stages. A review of 3244 citations identified 462 duplicates leaving 2767 citations. Titles and abstracts were then reviewed by two reviewers (BA and CJR) who applied the inclusion criteria. Any discrepancies were resolved through discussion. Full copies of the 32 papers were retained which met the inclusion criteria based on the abstract, of which 18 met the inclusion criteria for the review.

### Quality appraisal

Consideration of the quality of the empirical literature was not a central focus for inclusion of studies for the review. However, critical appraisal of strengths and weaknesses of included studies was necessary for the synthesis of literature. All of the studies identified were quantitative analytical surveys, where the checklist by Maltby *et al*. (2010) and CASP (Public Health Resource Unit 2006) were used to appraise methodological quality.

### Data extraction

A data extraction form was developed to help understand the features and strengths and weaknesses of included studies. The data extraction form was designed from early scrutiny of identified hand searched intentional research utilization articles. No scoring system was applied as the studies were not specifically chosen for their ‘quality’, but for their relevance to addressing the research question; thus, although some studies were weak methodologically, they were relevant. ‘Supplementary information Table S1’.

### Narrative data synthesis

The aim of data synthesis was to narratively interpret health professionals’ intentional research utilization behaviour. Narrative synthesis as described by Popay *et al*. (2006) was used to interpret and integrate quantitative primary empirical studies. These methods of synthesis can be used when included studies differ in health professional characteristics and quality, and when other integrative methods such as best evidence synthesis and meta‐analysis are not possible (Dixon‐Woods *et al*.^2004^).

Popay *et al*. (2006) describes four stages of synthesis (Supplementary information File S1). In this review, three of the four stages of synthesis (Popay *et al*. 2006) were used. The first stage involves developing a theory, which was not used as it was not a study objective. The following stages were used:

Stage 1: Developing a Preliminary Synthesis, this involved interpreting main outcomes of included studies.

Stage 2: Exploring the relationship within and between studies, this involved grouping studies with similar outcomes.

Stage 3: Assessing the Robustness of the synthesis, this involved reflecting on the value of synthesis methods in relation to the development of key findings.

#### Overview of the process

To help make sense of the range of data across included studies, tabulation and grouping and clustering were used. Tabulation produced a data extraction table to identify key characteristics across included studies (Supplementary information Table S1). The activity of grouping and clustering helped to identify similar results between studies. Using this approach, the main author and colleague firstly identified key outcomes (Supplementary information File S2). Then, key outcomes were condensed into groups, which involved interpreting the meaning of similar outcomes into groups (Supplementary information File S3). Braun and Clarke (2006) view the interpretation of key study results into themes as an inductive process of interpretation. This is judged to fit well with the development of themes in a narrative synthesis and is recommended to help summarize data as part of the thematic analysis (Popay *et al*. 2006). This process produced the following themes represented as groups:

Group 1: Theoretical intentional variables as dominant predictors of intention and intention of behaviour.

Group 2: Differences in how health professional groups form intentions.

Group 3: Competing explanations for the prediction of intention.

Study results in each group were then tabulated (Supplementary information Tables S2–S6). Tabulating results helped to identify the main quantitative results in each group. Five tables were developed that reflected the groups 1–3, Group 2 had two tables based on explaining behavioural differences by profession and behaviour.

## Results

### Theoretically based variables as dominant predictors of intention and intention of behaviour (Table S2)

#### Attitude

In intentional models attitude is either measured directly (a person's overall attitude, are they in favour of carrying out a behaviour) or indirectly often referred to as ‘behavioural belief’ (a person's beliefs which helps form an attitude) (Francis *et al*.^2004^). Attitudes are thought to influence intention and do not have a direct effect on behaviour (Ajzen 1991). Findings were discussed in relation to the dominant measure of attitude reported in each study.

Attitudes were a dominant predictor of intention in a range of behaviours, including infection control (Nurses’ glove use), providing educational advice (Practice Nurses’ Smoking cessation advice), antibiotic prescription (Surgical Physicians), delivering interventions (placing preventative fissure sealants) and assessment (C‐Spine and CT Head rules).

Nurses’ attitudes towards glove use were strong predictors of intention, wherein a positive attitude was thought to be mediated by the perceived risks of not wearing gloves – particularly when handling blood products (Watson & Myres 2001). Similarly, Practice Nurses demonstrated a positive attitude toward the planned delivery of new smoking cessation guidelines, when compared *t* = −7·36, *P* < 0·001 to Nurse Practitioners (Leitlen *et al*. 2011). However, Practice Nurses reported considerably more dissatisfaction with current guidelines when compared with Nurse Practitioners. Noted limitations of this study were the absence of reported response rate and a high number of missing questionnaire values (>20%) which were not appropriate for analysis (Leitlen *et al*. 2011).

Surgical Registrars reported a positive attitude towards using antibiotic guidelines, where the ‘usefulness’ of the guideline influenced their attitude. However, in this study, the increased focus on the measurement of ‘attitude’ (with increased number of items as opposed to others) may have resulted in the higher correlation *r* = 0·86 with attitude (Limbert & Lamb 2002). Physicians also demonstrated positive attitudes beta = 0·4, *P* < 0·001 towards the implementation of two decision‐aids C‐Spine Rule and CT Head Rules, although positive attitudes were only carried through beyond intention to actual behaviour for C‐Spine Rule (Perez *et al*. 2014). As suggested by Perez *et al*. (2014) constructs outside of TPB could explain CT Head Rules, as attitude as intentions were not carried through beyond intention into actual behaviour.

Dental Practitioners high intentions were significantly predicted by the belief of favourable outcomes Beta 0·29, *P* = 0·01 when simulating the placing preventative sealants (Bonetti *et al*. 2010). Similar beliefs about risk perception Beta 0·27, *P* = 0·01 and outcome expectancies Beta 0·30, *P* = 0·01, supported the predictive value of attitude‐based constructs with intention (Bonetti *et al*. 2010).

Establishing a pattern about how attitude influences intention is difficult, as intentions normally correspond to specific target behaviours (Francis *et al*. 2004). However, all attitude constructs indicated positive perceptions in reducing risk of cross‐infection (Watson & Myres 2001); improved educational guidance (Leitlen *et al*. 2011); usefulness of guidelines (Limbert & Lamb 2002); and reducing risk as a preventative intervention (Bonetti *et al*. 2010).

#### Subjective norm

Subjective norm (the influence of where and with whom you work) for physicians had varying effects on intention for different behaviours. Godin *et al*. (1998) reported that physicians were 14 times more likely (odds ratio) 14·61 *P* < 0·0001 to wear gloves as an expected professional behaviour, when in contact with blood or body fluids. Limbert and Lamb (2002) discovered that junior medical doctors expected to implement acute asthma guidelines were significantly influenced by professional colleagues *r* = 0·74, *P* < 0·001. By contrast, in the same study, senior doctors’ intentions were less influenced by professional colleagues, which perhaps could be an indication of greater professional autonomy or intellectual independence.

Anaesthetists (Beatty & Beatty 2004) intentions were significantly influenced by their normative beliefs (mean 67·9%) of violating pre‐ and postprocedure safety guidelines (pre‐op visits, cockpit checks, silencing alarms) (Beatty & Beatty 2004). Values of R2 indicated that subjective norm for all safety checks were statistically significant *P* < 0·05 and were robust enough to be examined as components for future intervention (Beatty & Beatty 2004). The sampling frame also indicated small demographic differences to the target population, despite the self‐selective sample (Beatty & Beatty 2004). Subjective norm, again was also a significant predictor of physicians’ intention *r* = 0·26, *P* < 0·001 when using assessment decision rules in the emergency department (Perez *et al*. 2014), which could suggest the influence of colleagues in specific departments.

Foy *et al*. (2005) also discovered that subjective norm was a powerful predictor of nurses’ intention when referring patients for an induced abortion (*r* = 0·52 *P* < 0·01). Subjective norm as in reaching professional agreement was an important motivator, despite personal beliefs. Similarly, Practice Nurses were 56·2% more likely to adopt new smoking cessation guidelines, partly influenced by their colleagues. However, in the same study this did not apply to Nurse Practitioners who had low intentions.

Kortteisto *et al*.'s (2010) Internet‐based cross‐sectional survey in Finland reported that Nurses’ intentions to use any type of patient‐specific guidelines in clinical decision‐making were mainly influenced by professional colleagues beta = 0·33, *P* < 0·001. Influence from professional colleagues also included other health professional groups, although their professions were not identified. Only 29% of the sample of Nurses responded which questions the representativeness of the sample.

Again, across behaviours it is difficult to pinpoint a pattern and the reasons as to why professional colleagues have an impact on intentions. Some studies indicate that in some departments and professional groups (Beatty & Beatty 2004, Perez *et al*. 2014) health professionals are more conscious of colleagues’ opinions and this has an effect on intention. Kortteisto *et al*. (2010) Internet‐based study suggests that nurses in Finland are significantly influenced by colleagues.

#### Perceived behavioural control

The Perceived Behavioural Control (PBC) and control beliefs represent the stated difficulty in performing behaviour (Ajzen 1991). The difficulty in performing guideline‐driven behaviour appears to be a key factor for nursing staff, where five of the seven studies report PBC as a significant predictor of intention across different types of behaviour, environment and grades of nurses.

Practice Nurses offering smoking cessation advice (guided by the National Service Framework), reported ‘time pressures’ as significant influences on intention when working to timed appointments (*r* = 0·546, *P* < 0·001) (Puffer & Rashidian 2004). O'Boyle *et al*. (2001) and Levin (1999) reported the problem of skin irritation for critical care nurses following hand‐washing guidelines, whilst the practicalities of cost (as a controlling factor) for sexual health nurses supplying contraceptives in an abortion clinic (*R* ^2^ 0·15) was also a significant predictor (Foy *et al*. 2005). These findings indicate that for some behaviours pragmatism determines intentional choice and outweighs the use of guideline‐driven evidence. Maue *et al*. (2004) study on guideline compliance also illustrated that advanced practice nurses perceived barriers *r* = −0·73, *P* < 0·0001 had a negative effect on intention. Although, the proportion of nurses in the sample is not clear and the same result applies to physicians.

Physicians’ intentions to implement general patient‐specific guidelines in clinical decision‐making were influenced by PBC beta 0·45, *P* < 0·001 (Kortteisto *et al*. 2010). Questionnaire items were enhanced by content derived from earlier studies and previous Finnish national documents (Kortteisto *et al*. 2010). This said elicitation studies were not conducted to help develop representative content for belief‐based questionnaire items. Buenestado *et al*. (2013) also established that the context where computerized asthma guidelines were implemented effects physicians intention *r* = 0·89, although the sample was limited to eight paediatricians.

#### Intention and the association with behaviour (Table S3)

Of the nine studies that investigated the relationship between intention and behaviour, seven studies measured self‐report behaviour. For nurses, intention was a significant predictor of self‐report behaviour in glove use *r* = 0·47, *P* < 0·01 (Levin 1999) *r* = 0·69, *P* < 0·01 (Watson & Myres 2001); adherence to hand hygiene guidelines *r* = 0·63, *P* < 0·0001 (O'Boyle *et al*. 2001) Beta 4·53, *P* < 0·001 (Jenner *et al*. 2002). Intention was not a significant predictor of general guideline use *r* = 0·13 for Advanced Nurse Practitioners (Maue *et al*. 2004).

For physicians, universal precautions to venepunctures *r* = 0·50, *P* < 0·0001 (Godin *et al*. 2000); adherence to asthma and antibiotic guidelines (Limbert & Lamb 2002) and adopting a C‐Spine Rule Odds Ratio 1·79, *P* < 0·01 were all positively associated with intention. Although, intention was not a significant predictor of general guideline use *r* = 0·13 for Physicians (Maue *et al*. 2004).

Mostly, the proportion of variance captured in these studies was over 28%, which is typical of the proportion of variance captured by intention (Godin *et al*. 2008). Although, studies have also shown that self‐report intentions are not always carried through to actual behaviour (O'Boyle *et al*. 2001). Thus, saying ‘X’ and doing ‘X’ cannot be relied on.

### Differences and similarities in how health professional groups form intentions

Differences in intentions across professional groups for the same behaviours were identified by organizing dominant predictors of intention by profession ‘Supplementary information Tables S4 and S5’.

Nurses and physicians form intentions in different ways when using gloves as guidance for infection control. Godin *et al*. (1998) identified that when wearing gloves is the accepted behaviour amongst physicians, there was a 14·61 greater odds of high intention to wear gloves. In this example, physicians’ behaviour could suggest that uniform behaviours in the medical profession are important and promote a strong subjective norm towards intention.

By contrast, nurses’ intentions towards wearing gloves can have different influences. Levin (1999) established that nurses’ and laboratory workers’ perceived control and attitude rather than subjective norm were key predictors of intention for glove use. Similarly, Watson and Myres (2001) established that attitudes (*R* ^2^ 0·63, *P* < 0·01) explained a large proportion of nurses’ glove use behaviour. These examples indicate that peer pressure and the working environment have different effects across professional groups when performing similar behaviours. However, this suggestion should be tempered because these studies were performed in different environments.

Differences in how nurses’ and physicians’ form intention were also identified for general guideline use. Kortteisto *et al*. (2010) highlighted that subjective norms were key determinates to general guideline use for Finnish nurses. In the same study, the dominant determinant for physicians was PBC (beta 0·45, *P* < 0·01) (Kortteisto *et al*. 2010).

Different determinants of intention are evident when nurses perform the same behaviour. O'Boyle *et al*. (2001) and Pessoa‐Silva *et al*. (2005) identified the perception of control and ability to perform hand hygiene important. Whereas, Jenner *et al*. (2002) reported the nurse's responsibility as a driving factor for intention.

By contrast, nurses and physicians often share similar determinants of intention for some instrumental research utilization behaviours. The usability or usefulness of a guideline can affect both the PBC and attitudes of nurses’ and physicians’ (Bolman *et al*. 2002, Limbert & Lamb 2002). These results indicate that the content and clarity of the guideline being used can have similar effects on how professionals’ form intentions.

### Competing explanations for the prediction of intention and behaviour

Competing explanations for the prediction of intention and behaviour are reported in ‘Supplementary information Table S6’. Competing explanations represent variables added to intentional models to explain their effect on intention, behaviour or both intention and behaviour. Some studies report the direct effect on intention other studies the effect on behaviour. Additional variables are added as previous research often has identified other variables which could explain health professionals’ intentions or behaviour in additional to theoretical model variables.

Eleven of the eighteen studies included in this review added variables to established theoretical models. Many of these variables were pragmatic and intertwined with the clinical behaviour. For example, O'Boyle *et al*. (2001) and Jenner *et al*. (2002) recognized that ‘time availability’, ‘intensity of activity’ and ‘the number and location of sinks’ could mediate health professionals’ hand‐washing behaviour and potentially override intentional choices. Likewise, other studies included personal factors related to the behaviour such as nurses own smoking behaviour when introducing smoking cessation guidelines (Bolman *et al*. 2002) and or personal responsibility in hand hygiene (Jenner *et al*. 2002) and factors related to the guideline itself such as perceived simplicity (Bolman *et al*. 2002) and satisfaction with current guidelines (Leitlen *et al*. 2011) when compared with the introduction of new guidelines.

Other variables were theory‐driven (habit) and recognized that behaviour is not always driven by intentional choices but by practiced behaviour (Beatty & Beatty 2004, Bonetti *et al*. 2010, Buenestado *et al*. 2013). Thus, there is increasing recognition that intentional behaviour is better understood by adding discrete (and relevant) variables for a more holistic understanding of intentional behaviour, for which there is some supporting evidence.

O'Boyle *et al*. (2001) identified that ‘observed intensity of activity’ interfered with intentions to comply with hand‐washing guidelines (*r* = −0·32, *P* < 0·05). O'Boyle *et al*. (2001) hypothesized that despite having good intentions to comply with hand hygiene practices (control beliefs), actual behaviour was influenced by the realities of clinical practice (O'Boyle *et al*. 2001). Perceived simplicity (*r* = 0·65, *P* < 0·01) was also the best predictor of intention when nurses were expected to use smoking cessation guidelines (Bolman *et al*. 2002). Jenner *et al*. (2002) also reported personal responsibility as the strongest predictor of intention (*r* = 0·42, *P* < 0·01) for compliance with hand hygiene. Thus, there is significant statistical support for the inclusion of additional variables to better understand intention.

Maue *et al*. (2004) identified that perceived barriers (*r* = −0·73, *P* < 0·0001) which constituted confidence; understanding and practice habits were negative contributors for general guideline compliance; external barriers (although not a dominant barrier) were also reported as significant (*r* = −0·47, *P* < 0·006). However, positive practice habits were significant in predicting dental practitioners intentions Beta = 0·59, *P* < 0·001 and behaviour Beta = 0·35, *P* < 0·001, where positive attitudes correlated with resulted practiced behaviours. Buenestado *et al*. (2013) also recognized that physicians’ uptake of asthma guidelines could be related to difficulties integrating into their actual practice.

However, not all ‘additional variables’ increase our understanding of intention, for example, demographic and skill‐based factors (age, clinical experience, patient demands) in this review did not report significant or much less statistical significant predictors of intention when compared with established social cognitive model variables (Godin *et al*. 1998, 2000, Puffer & Rashidian 2004).

Competing explanations for the prediction of intentions have also been evaluated by incrementally adding in ‘additional variables’ to discover their effect on intention and model determinates of intention. For example, Watson and Myres (2001) discovered that adding ‘perceived barriers’ to the PBC to understand glove use behaviour, increased the explanatory power of intention by 3·5%. Similarly, Levin (1999) reported that the explanatory power of variables in intentional models can change with the addition of other relevant variables.

From studies that measured additional variables to better understand intention and behaviour it can be concluded that this approach is helpful when:

☐ There is a careful selection of variables in respect to the behaviour under investigation
☐ To establish the additional benefits of adding variables to established model variables.

## Discussion

This study set out to understand health professionals’ intentional instrumental research utilization behaviour. The results helped to provide a platform for addressing the main *aims* of the review and review questions.

### Are there professional differences or similarities in intentional research utilization behaviours?

There do appear to be differences and also similarities in intentional research utilization behaviours across and in health professions. Making comparisons across professional groups is complicated by the range of professional groups identified and the limited number of comparable behaviours. However, the clearest comparisons can be made between nurses and physicians, as these have been the two main healthcare professional populations researched.

Nurses’ intentions to use gloves in clinical procedures, hand hygiene, smoking cessation and general guidelines are predominantly influenced by the perceived difficulty in performing the behaviour (PBC). These findings are supported by many studies where contextual factors are reported as important inhibitors of clinical guideline use (Rycroft‐Malone *et al*. 2009, Cummings *et al*. 2010, Schultz & Kitson 2010). Methods are now available to help researchers identify key variables for behaviour, where initial eliciting questions can help identify relevant variables for the specific behaviour under investigation (Michie *et al*. 2005). Dyson *et al*. (2013) recently used this approach and identified that the greater the number of barriers in hand hygiene practice the more this affected compliance.

It was also established that PBC and subjective norm variables influence nurses and physicians intentions differently. Across behaviours (glove use, hand hygiene, specialist guideline use) physicians were influenced by established peer practice (Godin *et al*. 1998, Limbert & Lamb 2002, Foy *et al*. 2005). By comparison, when nurses are faced with similar behaviours, the perceived difficulty in performing the behaviour is a key determinant of intention.

However, how intentions are formed can be similar across professional groups. Foy *et al*. (2005) identified subjective norm as a key determinate of intention across professional groups in abortion care. This indicates that for some behaviours, shared decision‐making is a key component in helping form intentions and is a process which complements clinical guideline decision‐making (Guerrier *et al*. 2013). In nursing practice decision‐making, protocol‐based care is most often viewed as a social activity (Rycroft‐Malone *et al*. 2009); which also indicates that the influence of professional colleagues' affects all professions, particularly in discrete clinical environments.

Professional differences in professions were also discovered. For example, UK nurse's general guideline use was influenced more by overcoming practical difficulties (as a predictor of intention), compared with nurses in Finland being influenced more by professional colleagues (Kortteisto *et al*. 2010). This indicates that contextual issues in terms of ‘usability’ and the influence of ‘leadership’ in influencing intentions could be an issue. The importance of leadership and usability of guidelines are recurring themes that have been identified as key influences in the implementation of clinical guidelines (Debourgh 2001, Chummun & Tiran 2008, Yousefi‐Nooraie *et al*. 2014).

### Are there any other influences on intentional behaviour in addition to those explained by model variables?

In this review it was highlighted that in many studies ‘additional variables’ are included to explain behaviour in addition to intentional model variables. It should be noted that for some authors ‘additional variables’ are seen as extensions of the PBC, whereas other authors see the same variables as being distinct from the PBC. Regardless, these variables do explain variations in intentional behaviour.

Additional variables appear to have most influence on behaviour when the behaviour is difficult to perform or behaviour is already established through practice ‘habit’ (Godin *et al*. 1998, Beatty & Beatty 2004, Maue *et al*. 2005, Bonetti *et al*. 2010) – which applies to all healthcare professionals.

It appears that habitual behaviour has an overriding effect on intention when behaviours are repeated. In this review, habit forming behaviours were evident in hand hygiene, glove use, pre‐operative visits and safety checks. Ouellette and Wood (1998) highlight that habitual behaviour occurs when there is a tendency to repeat past behaviours in a stable context, because the same contingencies are in place (Ouellette & Wood 1998). In this instance, behaviour is thought to come under control of stimulus cues and the presence of these cues triggers the automatic response sequence, bypassing cognitive processes such as attitude and intentions (Ouellette & Wood 1998). Godin *et al*. (2008) in their review of general health professional behaviour give empirical support to this theory, identifying habit as an important variable in the prediction of healthcare professionals’ behaviour.

Other variables explored alongside intentional variables were less predictive. The effect of demographic factors appears to have very little effect on intentional behaviour. Only one study reported a significant effect of ‘age’ as a contributor to intentional behaviour; and this had limited effects in comparison to the main effect of subjective norms (Godin *et al*. 1998). This type of finding upholds the theoretical structure of intentional models where demographic factors are not thought to directly influence intention (Ajzen 1991).

### In which circumstances is intention a powerful predictor of research utilization behaviour?

Half the studies in the review did not report the relationship between intention and behaviour. It is presumed that this is because intention is viewed as a proximal determinant of behaviour, and if we know the intention, then we know the likely behaviour. In this review, when the relationship between intention and behaviour was reported, there was a strong statistical relationship (Cohen 1988) between intention and self‐reported glove use, universal precautions to venepunctures, hand hygiene, antibiotic and asthma guidelines, general guideline use, placing fissure sealants in dental practice and use of decision rules.

Historically, across a range of behaviours, evidence suggests that intention can be a statistically reliable predictor of self‐report behaviour, predicting a good proportion of behaviour. Godin *et al*. (2008) explored a range of healthcare professional behaviours (one of which was guideline use) and proportioned a frequency weighted mean for intention of 59%. The findings of Godin *et al*. (2008) were consistent with previous reviews where the relative effectiveness of intention (as a predictive construct) in the Theory of Planned Behaviour also showed good predictive values – 40% and 33·7% respectively (Godin & Kok 1996, Conner & Sparks 2005).

Theoretical models of intention in this review included the Theory of Reasoned Action, Theory of Planned Behaviour, Theory of Interpersonal Behaviour, The Attitude, Social influence and Self‐efficacy Theory, The I‐Change Model and Technology Acceptance Model. Generally, increasing the number of variables increases the predictive value of intention. Given this, future use of intentional models should recognize the value of extending theoretical models to explain instrumental research use behaviour.

### Is there a consistent pattern of determinates of intentional research utilization behaviour?

Some potential patterns in terms of determinates of intention have emerged in relation to use of clinical guidelines. However, patterns only emerge in relation to certain behaviours, professional responses to certain behaviours and also in professional differences to similar behaviours across different countries. Therefore, it is suggested that healthcare professional intentional responses are driven by the behaviour and environment where the behaviour is performed, in which the determinates of intention appear to be intertwined within the context and realities of the health professional's role and professional circumstance.

Nurses’ perceived difficulties in carrying out guideline‐driven clinical behaviours (conceptualized by the PBC) appear as the dominant factor determining practical behaviours such as hand hygiene and glove use. Perceived difficulties also inhibit guideline use when using more complex guidelines (Puffer & Rashidian 2004). These examples of ‘perceived difficulties’ suggest that guidelines should be developed that take into consideration the nature of the nurse's role and usability of the guideline, which has been recognized as an important contextual factor (Rycroft‐Malone 2004, Brown *et al*. 2008, Chabot *et al*. 2010).

Professional differences in similar behaviours have also been highlighted (Kortteisto *et al*. 2010), which again demonstrates the influence of the professional environment where ‘role models’ and ‘peers’ do have an impact on intentions. It could be argued that professional behaviour is influenced (or emphasized) more by professional colleagues in other European countries; however, these comparisons are made with very few studies.

Additional variables do provide significant explanations particularly when the clinical environment and practice habits influence expected guideline‐driven behaviours. The measurement of habit is mostly confined to behaviours that are repeated and should be recognized when intentions are explored in clinical practice.

### Limitations

**Figure 1:**
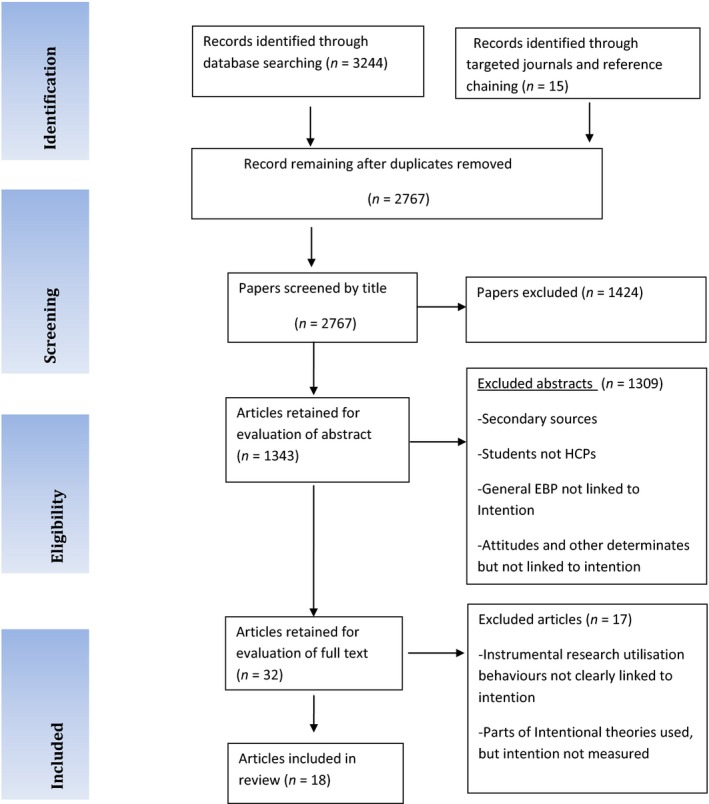
PRISMA flow diagram, based on inclusion and exclusion criteria.

This review set out to follow PRISMA guidelines (Moher 2009) and narrative synthesis (Popay *et al*. 2006) as a guide for the systematic review. All of the processes recommended were acknowledged; however, there are some weaknesses that could impact on the representativeness of the review outcomes (Figure 1).

The narrative synthesis methodology helped to organize key outcomes of the review. In this review key outcomes were represented by variables which reported the strongest association; other variables with a weaker association were not reported (despite being statistically significant). Nevertheless, this approach does identify the key associations between determinants of intention, intention and behaviour.

The evaluation of study quality focused on a general evaluation of potential moderator factors on reported outcomes (Supplementary information Table S1). In the process, some general methodological concerns across several studies and their influence on outcomes were identified; for example, the lack of elicitation studies to uncover beliefs and low response rates.

## Conclusions

This study is the first systematic review that has explored healthcare professionals’ intentional instrumental research utilization behaviour. As such this is an important first step in recognizing how intentions are formed and intentional decisions made by healthcare professionals in response to a variety of guideline‐driven behaviours.

Healthcare professionals responsible for implementing new guidelines should be aware of the dominant influences on health professionals’ intentions when recommending a guideline‐driven change in clinical practice. This review has established the professional role of the clinician in clinical practice as important, particularly in regard to nurses and physicians. Findings have indicated that nurses’ intentions are mostly influenced by their ability to carry out the guideline‐driven behaviour in the realities of the practice environment, whereas physicians’ intentions are often influenced by the usefulness and relevance of the guideline and the perceptions of peers.

The review has also highlighted that intentions and determinants of intentions often have less influence than practice habits, which can be facilitative or inhibitive for all health professionals. When ingrained in practice, habits can be the dominant driving force for behaviour (Jenner *et al*. 2002, Beatty & Beatty 2004, Bonetti *et al*. 2010, Buenestado *et al*. 2013). Therefore, when a change in practice is required it should be important to establish current practice behaviours alongside the intention to change practice.

Researchers should also recognize the best empirical approaches. Where possible it is recommended that comparisons should be drawn between intentional and actual behaviour and relevant additional variables (including context) should be measured to increase our understanding of intention. Given that contextual factors can vary across environments and that a limited number of clinical guideline behaviours have been explored (related to intention), this will help to provide focus for future research.

It should also be recognized that the intentions are behaviour‐specific and can change from one behaviour to the other. Some of the guideline‐driven behaviours in this review are similar and have produced similar cross‐professions intentions. There is an absence of exploring time‐specific repeated guideline behaviour, an opportunity for further research.

## Author contributions

All authors have agreed on the final version and meet at least one of the following criteria [recommended by the ICMJE (http://www.icmje.org/recommendations/)]:

- substantial contributions to conception and design, acquisition of data or analysis and interpretation of data;
- drafting the article or revising it critically for important intellectual content.

## Supporting information


**Data S1.** Database Search History (Supplementary Information File 1).

**File S1.** Options and Choices made for narrative synthesis (methods used highlighted in red).
**File S2.** Preliminary analysis: identifying main outcomes.
**File S3.** Grouping main outcomes.
**Table S1.**
**Table S2.** Theoretically based variables as dominant predictors of intention (Group 1).
**Table S3.** Studies which measured intention and the association with behaviour (Group 1).
**Table S4.** Differences and similarities in how Health Professionals form intentions (by behaviour) (Group 2).
**Table S5.** Differences in how professional groups form intentions (Group 2).
**Table S6.** Competing explanations for the prediction of intention and behaviour (Group 3).
