# Supplementary material for: What are health professionals’ intentions toward using research and products of research in clinical practice? A systematic review and narrative synthesis
Source: Nurs Open. 2015 Dec 17;3(3):125–39. doi: 10.1002/nop2.40 (PMC5047343; doi:10.1002/nop2.40)
Supplement: Supplementary file 1 — Data S1. Database Search History (Supplementary Information File 1). [file NOP2-3-125-s001.docx]

Database Search History (Supplementary Information File 1)

**CINAHL**

1. CINAHL; exp NURSES/; 119016 results.

2. CINAHL; nurs*.ti,ab; 282094 results.

3. CINAHL; exp NURSING PRACTICE/; 32487 results.

4. CINAHL; 1 OR 2 OR 3; 340808 results.

5. CINAHL; INTENTION/; 1066 results.

6. CINAHL; intent*.ti,ab; 11690 results.

7. CINAHL; exp ATTITUDE/; 151643 results.

8. CINAHL; NURSE ATTITUDES/; 13797 results.

9. CINAHL; exp ATTITUDE OF HEALTH PERSONNEL/; 35408 results.

10. CINAHL; ‘perceived social norm*’.ti,ab; 27 results.

11. CINAHL; SOCIAL ATTITUDES/; 2955 results.

12. CINAHL; BEHAVIORAL RESEARCH/ OR exp SOCIAL BEHAVIOR/; 9617 results.

13. CINAHL; PEER PRESSURE/; 400 results.

14. CINAHL; ‘social attitude*’.ti,ab; 92 results.

15. CINAHL; ‘social behaviour*’.ti,ab; 198 results.

16. CINAHL; ‘social behavior*’.ti,ab; 397 results.

17. CINAHL; (determinant* adj5 behaviour*).ti,ab; 201 results.

18. CINAHL; (determinant* adj5 behavior*).ti,ab; 393 results.

19. CINAHL; 5 OR 6 OR 7 OR 8 OR 9 OR 10 OR 11 OR 12 OR 13 OR 14 OR 15 OR 16 OR 17 OR 18; 168962

results.

20. CINAHL; RESEARCH, NURSING/ OR CLINICAL NURSING RESEARCH/ OR EDUCATION, NURSING,

RESEARCH-BASED/ OR NURSING PRACTICE, RESEARCH-BASED/; 18058 results.

21. CINAHL; (research adj5 utilis*).ti,ab; 245 results.

22. CINAHL; (research adj5 utiliz*).ti,ab; 1477 results.

23. CINAHL; (research adj5 transfer*).ti,ab; 237 results.

24. CINAHL; exp NURSING PRACTICE, EVIDENCE-BASED/ OR exp PROFESSIONAL PRACTICE,

EVIDENCE-BASED/; 26643 results.

25. CINAHL; (evidence ADJ based adj2 practice*).ti,ab; 5978 results.

26. CINAHL; 20 OR 21 OR 22 OR 23 OR 24 OR 25; 45205 results.

27. CINAHL; 4 AND 19 AND 26; 2048 results.

28. CINAHL; 27 [Limit to: (Language English)]; 1931 results.

29. CINAHL; exp MOTIVATION/; 28209 results.

30. CINAHL; 19 OR 29; 188817 results.

31. CINAHL; 4 AND 26 AND 30; 2362 results.

32. CINAHL; 31 [Limit to: (Language English)]; 2235 results.

Additional Search Terms: Guidelines, HCPs, Theories

1. CINAHL; exp NURSES/; 127737 results.

2. CINAHL; nurs*.ti,ab; 298279 results.

3. CINAHL; exp NURSING PRACTICE/; 35116 results.

4. CINAHL; 1 OR 2 OR 3; 361593 results.

5. CINAHL; INTENTION/; 1314 results.

6. CINAHL; intent*.ti,ab; 12911 results.

7. CINAHL; exp ATTITUDE/; 166293 results.

8. CINAHL; NURSE ATTITUDES/; 14920 results.

9. CINAHL; exp ATTITUDE OF HEALTH PERSONNEL/; 38604 results.

10. CINAHL; ‘perceived social norm*’.ti,ab; 31 results.

11. CINAHL; SOCIAL ATTITUDES/; 3362 results.

12. CINAHL; BEHAVIORAL RESEARCH/ OR exp SOCIAL BEHAVIOR/; 11151 results.

13. CINAHL; PEER PRESSURE/; 423 results.

14. CINAHL; ‘social attitude*’.ti,ab; 102 results.

15. CINAHL; ‘social behaviour*’.ti,ab; 232 results.

16. CINAHL; ‘social behavior*’.ti,ab; 436 results.

17. CINAHL; (determinant* adj5 behaviour*).ti,ab; 234 results.

18. CINAHL; (determinant* adj5 behavior*).ti,ab; 431 results.

19. CINAHL; 5 OR 6 OR 7 OR 8 OR 9 OR 10 OR 11 OR 12 OR 13 OR 14 OR 15 OR 16 OR 17 OR 18; 185847

results.

20. CINAHL; RESEARCH, NURSING/ OR CLINICAL NURSING RESEARCH/ OR EDUCATION, NURSING,

RESEARCH-BASED/ OR NURSING PRACTICE, RESEARCH-BASED/; 18839 results.

21. CINAHL; (research adj5 utilis*).ti,ab; 274 results.

22. CINAHL; (research adj5 utiliz*).ti,ab; 1554 results.

23. CINAHL; (research adj5 transfer*).ti,ab; 268 results.

24. CINAHL; exp NURSING PRACTICE, EVIDENCE-BASED/ OR exp PROFESSIONAL PRACTICE,

EVIDENCE-BASED/; 30119 results.

25. CINAHL; (evidence ADJ based adj2 practice*).ti,ab; 6733 results.

26. CINAHL; 20 OR 21 OR 22 OR 23 OR 24 OR 25; 49678 results.

27. CINAHL; 4 AND 19 AND 26; 2239 results.

28. CINAHL; 27 [Limit to: (Language English)]; 2109 results.

29. CINAHL; exp MOTIVATION/; 31448 results.

30. CINAHL; 19 OR 29; 207818 results.

31. CINAHL; 4 AND 26 AND 30; 2580 results.

32. CINAHL; 31 [Limit to: (Language English)]; 2439 results.

33. CINAHL; BANDURA'S SOCIAL COGNITIVE THEORY/; 825 results.

35. CINAHL; ‘Social learning theor*’.ti,ab; 210 results.

36. CINAHL; ‘Social cognitive theor*’.ti,ab; 439 results.

38. CINAHL; ‘Theory of planned behavior*’.ti,ab; 497 results.

39. CINAHL; ‘Theory of planned behaviour*’.ti,ab; 259 results.

40. CINAHL; exp PSYCHOLOGICAL THEORY/; 11626 results.

41. CINAHL; ‘Psychological theor*’.ti,ab; 175 results.

42. CINAHL; ‘Theory of reasoned action’.ti,ab; 263 results.

43. CINAHL; ‘Theory of interpersonal behaviour*’.ti,ab; 0 results.

45. CINAHL; (Social adj5 influence*).ti,ab; 2714 results.

46. CINAHL; ‘Theory of interpersonal behavior*’.ti,ab; 3 results.

47. CINAHL; (Self-efficacy adj5 model*).ti,ab; 301 results.

48. CINAHL; (Health ADJ belief adj5 model*).ti,ab; 796 results.

49. CINAHL; (Self-determination adj5 theor*).ti,ab; 123 results.

50. CINAHL; (Expectancy adj5 theor*).ti,ab; 59 results.

52. CINAHL; ‘Cognitive theor*’.ti,ab; 586 results.

53. CINAHL; ‘Locus of control’.ti,ab; 1268 results.

54. CINAHL; 33 OR 35 OR 36 OR 38 OR 39 OR 40 OR 41 OR 42 OR 43 OR 45 OR 46 OR 47 OR 48 OR 49 OR 50

OR 52 OR 53; 17024 results.

55. CINAHL; 19 AND 26 AND 54; 142 results.

56. CINAHL; 55 [Limit to: (Language English)]; 138 results.

58. CINAHL; exp HEALTH PERSONNEL/; 259656 results.

59. CINAHL; 58 not 4; 122371 results.

60. CINAHL; 19 AND 26 AND 59; 344 results.

NHS Evidence | library.nhs.uk

Page 7

61. CINAHL; 60 [Limit to: (Language English)]; 341 results.

62. CINAHL; GUIDELINE ADHERENCE/ [Limit to: (Language English)]; 1984 results.

63. CINAHL; 26 AND 62 [Limit to: (Language English)]; 240 results.

64. CINAHL; 63 [Limit to: (Language English) and (Language English)]; 240 results.

**MEDLINE**

69. MEDLINE; exp *’ATTITUDE OF HEALTH PERSONNEL’/; 49944 results.

70. MEDLINE; exp *SOCIAL BEHAVIOR/; 60817 results.

71. MEDLINE; exp INTENTION/; 4212 results.

75. MEDLINE; exp *MOTIVATION/; 43920 results.

76. MEDLINE; exp *ATTITUDE/; 114516 results.

77. MEDLINE; exp *BEHAVIORAL RESEARCH/; 1201 results.

78. MEDLINE; inten*.ti,ab; 504858 results.

79. MEDLINE; ‘perceived social norm*’.ti,ab; 70 results.

80. MEDLINE; (‘social behaviour’ OR ‘social behavior’).ti,ab; 4463 results.

81. MEDLINE; ‘social attitude*’.ti,ab; 442 results.

82. MEDLINE; ‘peer pressure*’.ti,ab; 596 results.

83. MEDLINE; (determinant* adj5 behaviour*).ti,ab; 690 results.

84. MEDLINE; (determinant* adj5 behavior*).ti,ab; 1561 results.

85. MEDLINE; 69 OR 70 OR 71 OR 75 OR 76 OR 77 OR 78 OR 79 OR 80 OR 81 OR 82 OR 83 OR 84; 723802

results.

87. MEDLINE; exp *NURSING RESEARCH/ OR exp *RESEARCH/; 157157 results.

88. MEDLINE; exp *EVIDENCE-BASED PRACTICE/; 16679 results.

89. MEDLINE; (research adj5 utilis*).ti,ab; 268 results.

90. MEDLINE; (research adj5 utiliz*).ti,ab; 2609 results.

91. MEDLINE; (evidence adj5 utiliz*).ti,ab; 1029 results.

92. MEDLINE; (evidence adj5 utilis*).ti,ab; 125 results.

93. MEDLINE; (engag* adj5 research).ti,ab; 1186 results.

94. MEDLINE; (engag* adj5 evidence).ti,ab; 266 results.

95. MEDLINE; (research adj5 transfer).ti,ab; 650 results.

NHS Evidence | library.nhs.uk

Page 7

96. MEDLINE; (evidence ADJ based ADJ practice*).ti,ab; 4208 results.

97. MEDLINE; 87 OR 88 OR 89 OR 90 OR 91 OR 92 OR 93 OR 94 OR 95 OR 96; 178796 results.

100. MEDLINE; nurs*.ti,ab; 287251 results.

101. MEDLINE; exp *NURSES/ OR exp *NURSING/; 163703 results.

102. MEDLINE; 100 OR 101; 360640 results.

103. MEDLINE; 85 AND 97 AND 102; 2175 results.

104. MEDLINE; 103 [Limit to: Humans and English Language]; 1972 results.

Additional Search Terms

1. MEDLINE; exp *’ATTITUDE OF HEALTH PERSONNEL’/; 52866 results.

2. MEDLINE; exp *SOCIAL BEHAVIOR/; 65623 results.

3. MEDLINE; exp INTENTION/; 4775 results.

4. MEDLINE; exp *MOTIVATION/; 46918 results.

5. MEDLINE; exp *ATTITUDE/; 121448 results.

6. MEDLINE; exp *BEHAVIORAL RESEARCH/; 1257 results.

7. MEDLINE; inten*.ti,ab; 540016 results.

8. MEDLINE; ‘perceived social norm*’.ti,ab; 79 results.

9. MEDLINE; (‘social behaviour’ OR ‘social behavior’).ti,ab; 4857 results.

10. MEDLINE; ‘social attitude*’.ti,ab; 477 results.

11. MEDLINE; ‘peer pressure*’.ti,ab; 643 results.

12. MEDLINE; (determinant* adj5 behaviour*).ti,ab; 757 results.

13. MEDLINE; (determinant* adj5 behavior*).ti,ab; 1670 results.

14. MEDLINE; 1 OR 2 OR 3 OR 4 OR 5 OR 6 OR 7 OR 8 OR 9 OR 10 OR 11 OR 12 OR 13; 773234 results.

15. MEDLINE; exp *NURSING RESEARCH/ OR exp *RESEARCH/; 210957 results.

16. MEDLINE; exp *EVIDENCE-BASED PRACTICE/; 18671 results.

17. MEDLINE; (research adj5 utilis*).ti,ab; 313 results.

18. MEDLINE; (research adj5 utiliz*).ti,ab; 2820 results.

19. MEDLINE; (evidence adj5 utiliz*).ti,ab; 1093 results.

20. MEDLINE; (evidence adj5 utilis*).ti,ab; 142 results.

21. MEDLINE; (engag* adj5 research).ti,ab; 1371 results.

22. MEDLINE; (engag* adj5 evidence).ti,ab; 329 results.

23. MEDLINE; (research adj5 transfer).ti,ab; 704 results.

24. MEDLINE; (evidence ADJ based ADJ practice*).ti,ab; 4850 results.

25. MEDLINE; 15 OR 16 OR 17 OR 18 OR 19 OR 20 OR 21 OR 22 OR 23 OR 24; 234395 results.

26. MEDLINE; nurs*.ti,ab; 300136 results.

27. MEDLINE; exp *NURSES/ OR exp *NURSING/; 170004 results.

28. MEDLINE; 26 OR 27; 375919 results.

29. MEDLINE; 14 AND 25 AND 28; 2621 results.

30. MEDLINE; 29 [Limit to: Humans and English Language]; 2391 results.

31. MEDLINE; exp PSYCHOLOGICAL THEORY/; 68119 results.

32. MEDLINE; ‘Social cognitive theor*’.ti,ab; 685 results.

33. MEDLINE; ‘Social learning theor*’.ti,ab; 417 results.

34. MEDLINE; ‘Theory of planned behaviour*’.ti,ab; 445 results.

35. MEDLINE; ‘Theory of planned behavior*’.ti,ab; 691 results.

36. MEDLINE; ‘Psychological theor*’.ti,ab; 783 results.

37. MEDLINE; ‘Theory of reasoned action’.ti,ab; 318 results.

40. MEDLINE; ‘Theory of interpersonal behaviour*’.ti,ab; 4 results.

41. MEDLINE; ‘Theory of interpersonal behavior*’.ti,ab; 4 results.

42. MEDLINE; (Social adj5 influence*).ti,ab; 7718 results.

43. MEDLINE; (Self-efficacy adj5 model*).ti,ab; 365 results.

44. MEDLINE; (Health ADJ belief adj5 model*).ti,ab; 1087 results.

45. MEDLINE; (Self-determination adj5 theor*).ti,ab; 287 results.

46. MEDLINE; (Expectancy adj5 theor*).ti,ab; 239 results.

47. MEDLINE; ‘Cognitive theor*’.ti,ab; 1387 results.

49. MEDLINE; ‘Locus of control’.ti,ab; 4600 results.

50. MEDLINE; 31 OR 32 OR 33 OR 34 OR 35 OR 36 OR 37 OR 40 OR 41 OR 42 OR 43 OR 44 OR 45 OR 46 OR 47

OR 49; 84338 results.

51. MEDLINE; 25 AND 50; 1118 results.

52. MEDLINE; 51 [Limit to: Humans and English Language]; 958 results.

53. MEDLINE; 14 AND 25 AND 50; 254 results.

54. MEDLINE; 53 [Limit to: Humans and English Language]; 227 results.

55. MEDLINE; exp HEALTH PERSONNEL/; 331746 results.

56. MEDLINE; 55 not 28; 228442 results.

57. MEDLINE; 14 AND 25 AND 56; 690 results.

58. MEDLINE; 57 [Limit to: Humans and English Language]; 600 results.

**PsycINFO**

105. PsycINFO; exp NURSES/; 16408 results.

106. PsycINFO; exp NURSING/; 9734 results.

107. PsycINFO; nurs*.ti,ab; 52279 results.

108. PsycINFO; 105 OR 106 OR 107; 53228 results.

109. PsycINFO; exp INTENTION/; 7196 results.

110. PsycINFO; exp ATTITUDES/ OR exp HEALTH PERSONNEL ATTITUDES/; 220483 results.

111. PsycINFO; exp MOTIVATION/; 48759 results.

112. PsycINFO; exp SOCIAL NORMS/; 4143 results.

113. PsycINFO; ‘perceived social norm*’.ti,ab; 104 results.

114. PsycINFO; exp SOCIAL BEHAVIOR/; 424501 results.

115. PsycINFO; ‘social attitude*’.ti,ab; 1680 results.

116. PsycINFO; (‘social behaviour*’ OR ‘social behavior’).ti,ab; 10376 results.

117. PsycINFO; exp PEER PRESSURE/; 456 results.

118. PsycINFO; (determinant* adj5 behaviour*).ti,ab; 364 results.

119. PsycINFO; (determinant* adj5 behavior*).ti,ab; 2502 results.

120. PsycINFO; (inten*).ti,ab; 142212 results.

121. PsycINFO; 109 OR 110 OR 111 OR 112 OR 113 OR 114 OR 115 OR 116 OR 117 OR 118 OR 119 OR 120;

742516 results.

122. PsycINFO; 108 AND 121; 18080 results.

123. PsycINFO; exp EVIDENCE BASED PRACTICE/; 6367 results.

124. PsycINFO; (evidence ADJ based adj5 practice*).ti,ab; 3996 results.

128. PsycINFO; (research adj5 utilis*).ti,ab; 121 results.

130. PsycINFO; (research adj5 utiliz*).ti,ab; 3109 results.

131. PsycINFO; (evidence adj5 utiliz*).ti,ab; 349 results.

132. PsycINFO; (evidence adj5 utilis).ti,ab; 0 results.

133. PsycINFO; (engag* adj5 research).ti,ab; 1758 results.

134. PsycINFO; (engag* adj5 evidence).ti,ab; 318 results.

135. PsycINFO; (research adj5 transfer).ti,ab; 429 results.

136. PsycINFO; exp EXPERIMENTATION/; 48341 results.

137. PsycINFO; 123 OR 124 OR 128 OR 130 OR 131 OR 132 OR 133 OR 134 OR 135 OR 136; 60373 results.

138. PsycINFO; 122 AND 137; 448 results.

139. PsycINFO; 138 [Limit to: English Language]; 446 results.

Additional Search Terms

1. PsycINFO; exp NURSES/; 17957 results.

2. PsycINFO; exp NURSING/; 10930 results.

3. PsycINFO; nurs*.ti,ab; 56669 results.

4. PsycINFO; 1 OR 2 OR 3; 57667 results.

5. PsycINFO; exp INTENTION/; 7923 results.

6. PsycINFO; exp ATTITUDES/ OR exp HEALTH PERSONNEL ATTITUDES/; 232329 results.

7. PsycINFO; exp MOTIVATION/; 51256 results.

8. PsycINFO; exp SOCIAL NORMS/; 4471 results.

9. PsycINFO; ‘perceived social norm*’.ti,ab; 113 results.

10. PsycINFO; exp SOCIAL BEHAVIOR/; 448338 results.

11. PsycINFO; ‘social attitude*’.ti,ab; 1742 results.

12. PsycINFO; (‘social behaviour*’ OR ‘social behavior’).ti,ab; 10944 results.

13. PsycINFO; exp PEER PRESSURE/; 481 results.

14. PsycINFO; (determinant* adj5 behaviour*).ti,ab; 399 results.

15. PsycINFO; (determinant* adj5 behavior*).ti,ab; 2617 results.

16. PsycINFO; (inten*).ti,ab; 153547 results.

17. PsycINFO; 5 OR 6 OR 7 OR 8 OR 9 OR 10 OR 11 OR 12 OR 13 OR 14 OR 15 OR 16; 786276 results.

18. PsycINFO; 4 AND 17; 19579 results.

19. PsycINFO; exp EVIDENCE BASED PRACTICE/; 7453 results.

20. PsycINFO; (evidence ADJ based adj5 practice*).ti,ab; 4722 results.

21. PsycINFO; (research adj5 utilis*).ti,ab; 144 results.

22. PsycINFO; (research adj5 utiliz*).ti,ab; 3429 results.

23. PsycINFO; (evidence adj5 utiliz*).ti,ab; 392 results.

24. PsycINFO; (evidence adj5 utilis).ti,ab; 0 results.

25. PsycINFO; (engag* adj5 research).ti,ab; 2042 results.

26. PsycINFO; (engag* adj5 evidence).ti,ab; 371 results.

27. PsycINFO; (research adj5 transfer).ti,ab; 478 results.

28. PsycINFO; exp EXPERIMENTATION/; 51593 results.

29. PsycINFO; 19 OR 20 OR 21 OR 22 OR 23 OR 24 OR 25 OR 26 OR 27 OR 28; 65478 results.

30. PsycINFO; 18 AND 29; 514 results.

31. PsycINFO; 30 [Limit to: English Language]; 511 results.

49. PsycINFO; exp SOCIAL COGNITION/ AND exp THEORIES/; 743 results.

50. PsycINFO; ‘Social cognitive theor*’.ti,ab; 1464 results.

51. PsycINFO; exp SOCIAL LEARNING/ AND exp LEARNING THEORY/; 182 results.

52. PsycINFO; ‘Social learning theor*’.ti,ab; 1804 results.

53. PsycINFO; exp PLANNED BEHAVIOR/ AND exp THEORIES/; 319 results.

54. PsycINFO; ‘Theory of planned behaviour*’.ti,ab; 520 results.

55. PsycINFO; ‘Theory of planned behavior*’.ti,ab; 1531 results.

56. PsycINFO; exp PSYCHOLOGICAL THEORIES/; 17158 results.

57. PsycINFO; ‘Psychological theor*’.ti,ab; 5996 results.

58. PsycINFO; exp REASONED ACTION/; 523 results.

59. PsycINFO; exp THEORIES/; 87799 results.

60. PsycINFO; 58 AND 59; 136 results.

61. PsycINFO; ‘Theory of reasoned action’.ti,ab; 879 results.

62. PsycINFO; ‘Theory of interpersonal behaviour*’.ti,ab; 2 results.

63. PsycINFO; ‘Theory of interpersonal behavior*’.ti,ab; 25 results.

64. PsycINFO; (Social adj5 influence*).ti,ab; 16472 results.

65. PsycINFO; (Self-efficacy adj5 model*).ti,ab; 804 results.

66. PsycINFO; (Health ADJ belief adj5 model*).ti,ab; 1069 results.

67. PsycINFO; (Self-determination adj5 theor*).ti,ab; 1116 results.

68. PsycINFO; (Expectancy adj5 theor*).ti,ab; 1189 results.

69. PsycINFO; exp COGNITIVE PSYCHOLOGY/ AND exp THEORIES/; 896 results.

70. PsycINFO; ‘Cognitive theor*’.ti,ab; 4282 results.

71. PsycINFO; exp ‘INTERNAL EXTERNAL LOCUS OF CONTROL’/; 12148 results.

72. PsycINFO; ‘Locus of control’.ti,ab; 12205 results.

73. PsycINFO; 49 OR 50 OR 51 OR 52 OR 53 OR 54 OR 55 OR 56 OR 57 OR 60 OR 61 OR 62 OR 63 OR 64 OR 65

OR 66 OR 67 OR 68 OR 69 OR 70 OR 71 OR 72; 66572 results.

74. PsycINFO; 17 AND 29 AND 73; 442 results.

75. PsycINFO; 74 [Limit to: Human and English Language]; 394 results.

NHS Evidence | library.nhs.uk

Page 9

77. PsycINFO; exp HEALTH PERSONNEL/; 86098 results.

78. PsycINFO; 77 not 4; 64919 results.

79. PsycINFO; 17 AND 29 AND 78; 443 results.

80. PsycINFO; 79 [Limit to: English Language]; 430 results.

81. PsycINFO; (guideline* adj3 adhere*).ti,ab; 404 results.

82. PsycINFO; (guideline* adj3 compliance*).ti,ab; 137 results.

83. PsycINFO; 81 OR 82; 530 results.

84. PsycINFO; 17 AND 29 AND 83; 12 results.

**BNI**

140. BNI; exp EVIDENCE BASED PRACTICE/; 1993 results.

142. BNI; (evidence ADJ based adj5 practice*).ti,ab; 870 results.

143. BNI; exp RESEARCH METHODS/; 4497 results.

144. BNI; (research adj5 utilis*).ti,ab; 103 results.

145. BNI; (research adj5 utiliz*).ti,ab; 167 results.

146. BNI; (evidence adj5 utiliz*).ti,ab; 8 results.

147. BNI; (evidence adj5 utilis*).ti,ab; 18 results.

148. BNI; (research adj5 transfer).ti,ab; 25 results.

149. BNI; (engag* adj5 research).ti,ab; 37 results.

150. BNI; (engag* adj5 evidence).ti,ab; 8 results.

151. BNI; 140 OR 142 OR 143 OR 144 OR 145 OR 146 OR 147 OR 148 OR 149 OR 150; 6675 results.

152. BNI; inten*.ti,ab; 3983 results.

153. BNI; motivat*.ti,ab; 961 results.

154. BNI; exp STAFF : ATTITUDES/; 3193 results.

155. BNI; attitude*.ti,ab; 5551 results.

156. BNI; (‘social behaviour’ OR ‘social behavior’).ti,ab; 28 results.

157. BNI; ‘peer pressure*’.ti,ab; 14 results.

158. BNI; ‘perceived social norm*’.ti,ab; 1 results.

159. BNI; (determinant* adj5 behaviour*).ti,ab; 21 results.

160. BNI; (determinant* adj5 behavior*).ti,ab; 12 results.

161. BNI; 152 OR 153 OR 154 OR 155 OR 156 OR 157 OR 158 OR 159 OR 160; 12199 results.

NHS Evidence | library.nhs.uk

Page 8

162. BNI; 151 AND 161; 305 results.

163. BNI; exp STUDENT NURSES/ OR exp STAFF NURSES/; 2396 results.

164. BNI; exp NURSING/; 59869 results.

165. BNI; nurs*.ti,ab; 80743 results.

166. BNI; 162 AND 165; 180 results.

Additional Search Terms

1. BNI; exp EVIDENCE BASED PRACTICE/; 2123 results.

2. BNI; (evidence ADJ based adj5 practice*).ti,ab; 965 results.

3. BNI; exp RESEARCH METHODS/; 4766 results.

4. BNI; (research adj5 utilis*).ti,ab; 113 results.

5. BNI; (research adj5 utiliz*).ti,ab; 169 results.

6. BNI; (evidence adj5 utiliz*).ti,ab; 8 results.

7. BNI; (evidence adj5 utilis*).ti,ab; 20 results.

8. BNI; (research adj5 transfer).ti,ab; 30 results.

9. BNI; (engag* adj5 research).ti,ab; 46 results.

10. BNI; (engag* adj5 evidence).ti,ab; 11 results.

11. BNI; 1 OR 2 OR 3 OR 4 OR 5 OR 6 OR 7 OR 8 OR 9 OR 10; 7111 results.

12. BNI; inten*.ti,ab; 4223 results.

13. BNI; motivat*.ti,ab; 1078 results.

14. BNI; exp STAFF : ATTITUDES/; 3599 results.

15. BNI; attitude*.ti,ab; 5969 results.

16. BNI; (‘social behaviour’ OR ‘social behavior’).ti,ab; 31 results.

17. BNI; ‘peer pressure*’.ti,ab; 15 results.

18. BNI; ‘perceived social norm*’.ti,ab; 1 results.

19. BNI; (determinant* adj5 behaviour*).ti,ab; 21 results.

20. BNI; (determinant* adj5 behavior*).ti,ab; 12 results.

21. BNI; 12 OR 13 OR 14 OR 15 OR 16 OR 17 OR 18 OR 19 OR 20; 13219 results.

22. BNI; 11 AND 21; 341 results.

23. BNI; exp STUDENT NURSES/ OR exp STAFF NURSES/; 2631 results.

24. BNI; exp NURSING/; 63082 results.

25. BNI; nurs*.ti,ab; 84592 results.

26. BNI; 22 AND 25; 204 results.

27. BNI; exp MEDICAL PROFESSION/; 4421 results.

28. BNI; exp PARAMEDICAL PROFESSIONS/; 1972 results.

29. BNI; exp PHARMACISTS/ OR exp PHYSIOTHERAPY/; 1034 results.

30. BNI; exp OCCUPATIONAL THERAPY/; 300 results.

31. BNI; exp DENTISTRY/ OR exp HEALTH VISITING/; 2073 results.

32. BNI; ‘allied health profession*’.ti,ab; 111 results.

33. BNI; 27 OR 28 OR 29 OR 30 OR 31 OR 32; 8501 results.

34. BNI; 33 not 26; 8498 results.

35. BNI; 11 AND 21 AND 34; 2 results.

36. BNI; exp ‘MODELS AND THEORIES’/; 5398 results.

37. BNI; ‘PSYCHOLOGICAL THEOR*’.ti,ab; 14 results.

38. BNI; ‘Social cognitive theor*’.ti,ab; 21 results.

39. BNI; ‘Social learning theor*’.ti,ab; 18 results.

40. BNI; ‘Theory of planned behaviour*’.ti,ab; 56 results.

41. BNI; ‘Theory of planned behavior*’.ti,ab; 26 results.

42. BNI; ‘Psychological theor*’.ti,ab; 14 results.

43. BNI; ‘Theory of reasoned action’.ti,ab; 26 results.

44. BNI; ‘Theory of interpersonal behaviour*’.ti,ab; 0 results.

45. BNI; ‘Theory of interpersonal behavior*’.ti,ab; 0 results.

46. BNI; (Social adj5 influence*).ti,ab; 179 results.

47. BNI; (Self-efficacy adj5 model*).ti,ab; 14 results.

48. BNI; (Health ADJ belief adj5 model*).ti,ab; 111 results.

49. BNI; (Self-determination adj5 theor*).ti,ab; 8 results.

50. BNI; (Expectancy adj5 theor*).ti,ab; 3 results.

51. BNI; ‘Cognitive theor*’.ti,ab; 27 results.

52. BNI; ‘Locus of control’.ti,ab; 120 results.

53. BNI; 37 OR 38 OR 39 OR 40 OR 41 OR 42 OR 43 OR 44 OR 45 OR 46 OR 47 OR 48 OR 49 OR 50 OR 51 OR

52; 571 results.

54. BNI; 11 AND 21 AND 53; 3 results.

55. BNI; (guideline* adj3 adhere*).ti,ab; 55 results.

56. BNI; (guideline* adj3 compliance*).ti,ab; 50 results.

NHS Evidence | library.nhs.uk

Page 3

57. BNI; 55 OR 56; 103 results.

58. BNI; 11 AND 57; 8 results.

Plus HCPs

1. BNI; exp EVIDENCE BASED PRACTICE/; 2123 results.

2. BNI; (evidence ADJ based adj5 practice*).ti,ab; 965 results.

3. BNI; exp RESEARCH METHODS/; 4766 results.

4. BNI; (research adj5 utilis*).ti,ab; 113 results.

5. BNI; (research adj5 utiliz*).ti,ab; 169 results.

6. BNI; (evidence adj5 utiliz*).ti,ab; 8 results.

7. BNI; (evidence adj5 utilis*).ti,ab; 20 results.

8. BNI; (research adj5 transfer).ti,ab; 30 results.

9. BNI; (engag* adj5 research).ti,ab; 46 results.

10. BNI; (engag* adj5 evidence).ti,ab; 11 results.

11. BNI; 1 OR 2 OR 3 OR 4 OR 5 OR 6 OR 7 OR 8 OR 9 OR 10; 7111 results.

12. BNI; inten*.ti,ab; 4223 results.

13. BNI; motivat*.ti,ab; 1078 results.

14. BNI; exp STAFF : ATTITUDES/; 3599 results.

15. BNI; attitude*.ti,ab; 5969 results.

16. BNI; (‘social behaviour’ OR ‘social behavior’).ti,ab; 31 results.

17. BNI; ‘peer pressure*’.ti,ab; 15 results.

18. BNI; ‘perceived social norm*’.ti,ab; 1 results.

19. BNI; (determinant* adj5 behaviour*).ti,ab; 21 results.

20. BNI; (determinant* adj5 behavior*).ti,ab; 12 results.

21. BNI; 12 OR 13 OR 14 OR 15 OR 16 OR 17 OR 18 OR 19 OR 20; 13219 results.

22. BNI; 11 AND 21; 341 results.

23. BNI; exp STUDENT NURSES/ OR exp STAFF NURSES/; 2631 results.

24. BNI; exp NURSING/; 63082 results.

25. BNI; nurs*.ti,ab; 84592 results.

26. BNI; 22 AND 25; 204 results.

27. BNI; exp MEDICAL PROFESSION/; 4421 results.

28. BNI; exp PARAMEDICAL PROFESSIONS/; 1972 results.

29. BNI; exp PHARMACISTS/ OR exp PHYSIOTHERAPY/; 1034 results.

30. BNI; exp OCCUPATIONAL THERAPY/; 300 results.

31. BNI; exp DENTISTRY/ OR exp HEALTH VISITING/; 2073 results.

32. BNI; ‘allied health profession*’.ti,ab; 111 results.

33. BNI; 27 OR 28 OR 29 OR 30 OR 31 OR 32; 8501 results.

34. BNI; 33 not 26; 8498 results.

35. BNI; 11 AND 21 AND 34; 2 results.

Plus Guidelines

1. BNI; exp EVIDENCE BASED PRACTICE/; 2123 results.

2. BNI; (evidence ADJ based adj5 practice*).ti,ab; 965 results.

3. BNI; exp RESEARCH METHODS/; 4766 results.

4. BNI; (research adj5 utilis*).ti,ab; 113 results.

5. BNI; (research adj5 utiliz*).ti,ab; 169 results.

6. BNI; (evidence adj5 utiliz*).ti,ab; 8 results.

7. BNI; (evidence adj5 utilis*).ti,ab; 20 results.

8. BNI; (research adj5 transfer).ti,ab; 30 results.

9. BNI; (engag* adj5 research).ti,ab; 46 results.

10. BNI; (engag* adj5 evidence).ti,ab; 11 results.

11. BNI; 1 OR 2 OR 3 OR 4 OR 5 OR 6 OR 7 OR 8 OR 9 OR 10; 7111 results.

12. BNI; inten*.ti,ab; 4223 results.

13. BNI; motivat*.ti,ab; 1078 results.

14. BNI; exp STAFF : ATTITUDES/; 3599 results.

15. BNI; attitude*.ti,ab; 5969 results.

16. BNI; (‘social behaviour’ OR ‘social behavior’).ti,ab; 31 results.

17. BNI; ‘peer pressure*’.ti,ab; 15 results.

18. BNI; ‘perceived social norm*’.ti,ab; 1 results.

19. BNI; (determinant* adj5 behaviour*).ti,ab; 21 results.

20. BNI; (determinant* adj5 behavior*).ti,ab; 12 results.

21. BNI; 12 OR 13 OR 14 OR 15 OR 16 OR 17 OR 18 OR 19 OR 20; 13219 results.

22. BNI; 11 AND 21; 341 results.

23. BNI; exp STUDENT NURSES/ OR exp STAFF NURSES/; 2631 results.

24. BNI; exp NURSING/; 63082 results.

25. BNI; nurs*.ti,ab; 84592 results.

26. BNI; 22 AND 25; 204 results.

27. BNI; exp MEDICAL PROFESSION/; 4421 results.

28. BNI; exp PARAMEDICAL PROFESSIONS/; 1972 results.

29. BNI; exp PHARMACISTS/ OR exp PHYSIOTHERAPY/; 1034 results.

30. BNI; exp OCCUPATIONAL THERAPY/; 300 results.

31. BNI; exp DENTISTRY/ OR exp HEALTH VISITING/; 2073 results.

32. BNI; ‘allied health profession*’.ti,ab; 111 results.

33. BNI; 27 OR 28 OR 29 OR 30 OR 31 OR 32; 8501 results.

34. BNI; 33 not 26; 8498 results.

35. BNI; 11 AND 21 AND 34; 2 results.

36. BNI; exp ‘MODELS AND THEORIES’/; 5398 results.

37. BNI; ‘PSYCHOLOGICAL THEOR*’.ti,ab; 14 results.

38. BNI; ‘Social cognitive theor*’.ti,ab; 21 results.

39. BNI; ‘Social learning theor*’.ti,ab; 18 results.

40. BNI; ‘Theory of planned behaviour*’.ti,ab; 56 results.

41. BNI; ‘Theory of planned behavior*’.ti,ab; 26 results.

42. BNI; ‘Psychological theor*’.ti,ab; 14 results.

43. BNI; ‘Theory of reasoned action’.ti,ab; 26 results.

44. BNI; ‘Theory of interpersonal behaviour*’.ti,ab; 0 results.

45. BNI; ‘Theory of interpersonal behavior*’.ti,ab; 0 results.

46. BNI; (Social adj5 influence*).ti,ab; 179 results.

47. BNI; (Self-efficacy adj5 model*).ti,ab; 14 results.

48. BNI; (Health ADJ belief adj5 model*).ti,ab; 111 results.

49. BNI; (Self-determination adj5 theor*).ti,ab; 8 results.

50. BNI; (Expectancy adj5 theor*).ti,ab; 3 results.

51. BNI; ‘Cognitive theor*’.ti,ab; 27 results.

52. BNI; ‘Locus of control’.ti,ab; 120 results.

53. BNI; 37 OR 38 OR 39 OR 40 OR 41 OR 42 OR 43 OR 44 OR 45 OR 46 OR 47 OR 48 OR 49 OR 50 OR 51 OR

52; 571 results.

54. BNI; 11 AND 21 AND 53; 3 results.

55. BNI; (guideline* adj3 adhere*).ti,ab; 55 results.

56. BNI; (guideline* adj3 compliance*).ti,ab; 50 results.

NHS Evidence | library.nhs.uk

Page 3

57. BNI; 55 OR 56; 103 results.

58. BNI; 11 AND 57; 8 results.

Plus Theories

1. BNI; exp EVIDENCE BASED PRACTICE/; 2123 results.

2. BNI; (evidence ADJ based adj5 practice*).ti,ab; 965 results.

3. BNI; exp RESEARCH METHODS/; 4766 results.

4. BNI; (research adj5 utilis*).ti,ab; 113 results.

5. BNI; (research adj5 utiliz*).ti,ab; 169 results.

6. BNI; (evidence adj5 utiliz*).ti,ab; 8 results.

7. BNI; (evidence adj5 utilis*).ti,ab; 20 results.

8. BNI; (research adj5 transfer).ti,ab; 30 results.

9. BNI; (engag* adj5 research).ti,ab; 46 results.

10. BNI; (engag* adj5 evidence).ti,ab; 11 results.

11. BNI; 1 OR 2 OR 3 OR 4 OR 5 OR 6 OR 7 OR 8 OR 9 OR 10; 7111 results.

12. BNI; inten*.ti,ab; 4223 results.

13. BNI; motivat*.ti,ab; 1078 results.

14. BNI; exp STAFF : ATTITUDES/; 3599 results.

15. BNI; attitude*.ti,ab; 5969 results.

16. BNI; (‘social behaviour’ OR ‘social behavior’).ti,ab; 31 results.

17. BNI; ‘peer pressure*’.ti,ab; 15 results.

18. BNI; ‘perceived social norm*’.ti,ab; 1 results.

19. BNI; (determinant* adj5 behaviour*).ti,ab; 21 results.

20. BNI; (determinant* adj5 behavior*).ti,ab; 12 results.

21. BNI; 12 OR 13 OR 14 OR 15 OR 16 OR 17 OR 18 OR 19 OR 20; 13219 results.

22. BNI; 11 AND 21; 341 results.

23. BNI; exp STUDENT NURSES/ OR exp STAFF NURSES/; 2631 results.

24. BNI; exp NURSING/; 63082 results.

25. BNI; nurs*.ti,ab; 84592 results.

26. BNI; 22 AND 25; 204 results.

27. BNI; exp MEDICAL PROFESSION/; 4421 results.

28. BNI; exp PARAMEDICAL PROFESSIONS/; 1972 results.

29. BNI; exp PHARMACISTS/ OR exp PHYSIOTHERAPY/; 1034 results.

30. BNI; exp OCCUPATIONAL THERAPY/; 300 results.

31. BNI; exp DENTISTRY/ OR exp HEALTH VISITING/; 2073 results.

32. BNI; ‘allied health profession*’.ti,ab; 111 results.

33. BNI; 27 OR 28 OR 29 OR 30 OR 31 OR 32; 8501 results.

34. BNI; 33 not 26; 8498 results.

35. BNI; 11 AND 21 AND 34; 2 results.

36. BNI; exp ‘MODELS AND THEORIES’/; 5398 results.

37. BNI; ‘PSYCHOLOGICAL THEOR*’.ti,ab; 14 results.

38. BNI; ‘Social cognitive theor*’.ti,ab; 21 results.

39. BNI; ‘Social learning theor*’.ti,ab; 18 results.

40. BNI; ‘Theory of planned behaviour*’.ti,ab; 56 results.

41. BNI; ‘Theory of planned behavior*’.ti,ab; 26 results.

42. BNI; ‘Psychological theor*’.ti,ab; 14 results.

43. BNI; ‘Theory of reasoned action’.ti,ab; 26 results.

44. BNI; ‘Theory of interpersonal behaviour*’.ti,ab; 0 results.

45. BNI; ‘Theory of interpersonal behavior*’.ti,ab; 0 results.

46. BNI; (Social adj5 influence*).ti,ab; 179 results.

47. BNI; (Self-efficacy adj5 model*).ti,ab; 14 results.

48. BNI; (Health ADJ belief adj5 model*).ti,ab; 111 results.

49. BNI; (Self-determination adj5 theor*).ti,ab; 8 results.

50. BNI; (Expectancy adj5 theor*).ti,ab; 3 results.

51. BNI; ‘Cognitive theor*’.ti,ab; 27 results.

52. BNI; ‘Locus of control’.ti,ab; 120 results.

53. BNI; 37 OR 38 OR 39 OR 40 OR 41 OR 42 OR 43 OR 44 OR 45 OR 46 OR 47 OR 48 OR 49 OR 50 OR 51 OR

52; 571 results.

54. BNI; 11 AND 21 AND 53; 3 results.

**HMIC**

167. HMIC; exp EVIDENCE BASED PRACTICE/; 792 results.

168. HMIC; (evidence ADJ based adj5 practice*).ti,ab; 1141 results.

169. HMIC; exp RESEARCH/; 6489 results.

170. HMIC; (research adj5 utilis*).ti,ab; 233 results.

171. HMIC; (research adj5 utiliz*).ti,ab; 59 results.

172. HMIC; (evidence adj5 utiliz*).ti,ab; 20 results.

173. HMIC; (evidence adj5 utilis*).ti,ab; 79 results.

174. HMIC; (research adj5 transfer).ti,ab; 46 results.

175. HMIC; (engag* adj5 research).ti,ab; 131 results.

176. HMIC; (engag* adj5 evidence).ti,ab; 54 results.

177. HMIC; 167 OR 168 OR 169 OR 170 OR 171 OR 172 OR 173 OR 174 OR 175 OR 176; 8325 results.

178. HMIC; exp NURSES/; 11290 results.

179. HMIC; exp NURSING/; 9141 results.

180. HMIC; nurs*.ti,ab; 30133 results.

181. HMIC; 178 OR 179 OR 180; 35700 results.

182. HMIC; 177 AND 181; 2120 results.

183. HMIC; inten*.ti,ab; 7788 results.

184. HMIC; exp MOTIVATION/; 503 results.

185. HMIC; exp ATTITUDES/ OR exp STAFF ATTITUDES/; 8078 results.

186. HMIC; ‘social attitude*’.ti,ab; 115 results.

187. HMIC; exp SOCIAL BEHAVIOUR/; 99 results.

188. HMIC; (‘social behaviour’ OR ‘social behavior’).ti,ab; 93 results.

189. HMIC; ‘peer pressure*’.ti,ab; 45 results.

190. HMIC; ‘perceived social norm*’.ti,ab; 3 results.

191. HMIC; (determinant* adj5 behaviour*).ti,ab; 71 results.

192. HMIC; (determinant* adj5 behavior*).ti,ab; 1 results.

193. HMIC; 183 OR 184 OR 185 OR 186 OR 187 OR 188 OR 189 OR 190 OR 191 OR 192; 16327 results.

194. HMIC; 182 AND 193; 284 results.

195. BNI,HMIC; Duplicate filtered: [162 AND 165], [182 AND 193]; 464 results

Additional Searches: Guidelines, HCPs, Theories

1. HMIC; exp EVIDENCE BASED PRACTICE/; 1995 results.

2. HMIC; (evidence ADJ based adj5 practice*).ti,ab; 1202 results.

3. HMIC; exp RESEARCH/; 8408 results.

4. HMIC; (research adj5 utilis*).ti,ab; 195 results.

5. HMIC; (research adj5 utiliz*).ti,ab; 64 results.

6. HMIC; (evidence adj5 utiliz*).ti,ab; 15 results.

7. HMIC; (evidence adj5 utilis*).ti,ab; 68 results.

8. HMIC; (research adj5 transfer).ti,ab; 30 results.

9. HMIC; (engag* adj5 research).ti,ab; 125 results.

10. HMIC; (engag* adj5 evidence).ti,ab; 38 results.

11. HMIC; 1 OR 2 OR 3 OR 4 OR 5 OR 6 OR 7 OR 8 OR 9 OR 10; 10958 results.

12. HMIC; exp NURSES/; 18137 results.

13. HMIC; exp NURSING/; 13701 results.

14. HMIC; nurs*.ti,ab; 34798 results.

15. HMIC; 12 OR 13 OR 14; 42629 results.

16. HMIC; 11 AND 15; 2739 results.

17. HMIC; inten*.ti,ab; 9668 results.

18. HMIC; exp MOTIVATION/; 870 results.

19. HMIC; exp ATTITUDES/ OR exp STAFF ATTITUDES/; 16251 results.

20. HMIC; ‘social attitude*’.ti,ab; 117 results.

21. HMIC; exp SOCIAL BEHAVIOUR/; 152 results.

22. HMIC; (‘social behaviour’ OR ‘social behavior’).ti,ab; 110 results.

23. HMIC; ‘peer pressure*’.ti,ab; 50 results.

24. HMIC; ‘perceived social norm*’.ti,ab; 6 results.

25. HMIC; (determinant* adj5 behaviour*).ti,ab; 83 results.

26. HMIC; (determinant* adj5 behavior*).ti,ab; 0 results.

27. HMIC; 17 OR 18 OR 19 OR 20 OR 21 OR 22 OR 23 OR 24 OR 25 OR 26; 26458 results.

28. HMIC; 16 AND 27; 364 results.

29. HMIC; exp PSYCHOLOGICAL THEORY/; 23 results.

30. HMIC; ‘Social cognitive theor*’.ti,ab; 14 results.

31. HMIC; ‘Social learning theor*’.ti,ab; 12 results.

32. HMIC; ‘Theory of planned behaviour*’.ti,ab; 64 results.

33. HMIC; ‘Theory of planned behavior*’.ti,ab; 2 results.

34. HMIC; ‘Psychological theor*’.ti,ab; 52 results.

35. HMIC; ‘Theory of reasoned action’.ti,ab; 12 results.

36. HMIC; ‘Theory of interpersonal behaviour*’.ti,ab; 0 results.

37. HMIC; ‘Theory of interpersonal behavior*’.ti,ab; 0 results.

38. HMIC; (Social adj5 influence*).ti,ab; 521 results.

39. HMIC; (Self-efficacy adj5 model*).ti,ab; 16 results.

40. HMIC; (Health ADJ belief adj5 model*).ti,ab; 49 results.

41. HMIC; (Self-determination adj5 theor*).ti,ab; 5 results.

42. HMIC; (Expectancy adj5 theor*).ti,ab; 4 results.

43. HMIC; ‘Cognitive theor*’.ti,ab; 20 results.

44. HMIC; ‘Locus of control’.ti,ab; 104 results.

45. HMIC; 29 OR 30 OR 31 OR 32 OR 33 OR 34 OR 35 OR 36 OR 37 OR 38 OR 39 OR 40 OR 41 OR 42 OR 43 OR

44; 831 results.

46. HMIC; 11 AND 27 AND 45; 4 results.

47. HMIC; exp HEALTH SERVICE STAFF/; 46910 results.

48. HMIC; 47 not 15; 27236 results.

49. HMIC; 11 AND 27 AND 48; 90 results.

50. HMIC; (guideline* adj3 adhere*).ti,ab; 116 results.

51. HMIC; (guideline* adj3 compliance*).ti,ab; 90 results.

52. HMIC; 50 OR 51; 203 results.

53. HMIC; 11 AND 27 AND 52; 3 results.
