# Supplementary material for: What are health professionals’ intentions toward using research and products of research in clinical practice? A systematic review and narrative synthesis
Source: Nurs Open. 2015 Dec 17;3(3):125–39. doi: 10.1002/nop2.40 (PMC5047343; doi:10.1002/nop2.40)
Supplement: Supplementary file 2 — File S1. Options and Choices made for narrative synthesis (methods used highlighted in red). File S2. Preliminary analysis: identifying main outcomes. File S3. Grouping main outcomes. Table S1. Table S2. Theoretically based variables as dominant predictors of intention (Group 1). Table S3. Studies which measured intention and the association with behaviour (Group 1). Table S4. Differences and similarities in how Health Professionals form intentions (by behaviour) (Group 2). Table S5. Differences in how professional groups form intentions (Group 2). Table S6. Competing explanations for the prediction of intention and behaviour (Group 3). [file NOP2-3-125-s002.docx]

Supplementary Information File 1: Options and Choices made for narrative synthesis (methods used highlighted in red)

*Not applicable*

*Not applicable*

**Assessing the robustness of the synthesis**

-Moderator variables and subgroup analysis

-Idea webbing and concept mapping

-Qualitative case descriptions

-Visual representation of relationship between study characteristics and results

**Exploring relationships within and between studies**

*Not applicable*

*Not applicable*

-Tabulation

-Grouping

-Transforming data

Conclusions and Recommendations

-Best evidence synthesis

-Checking the synthesis with authors of primary studies

-Conceptual triangulation

-Reciprocal translation

-Investigator and methodological translation

-Textual descriptions

-Translating data

**Developing a preliminary synthesis**

**Developing a theory**

18 studies

- Reflecting critically on the synthesis process

| **Author and Title** | **Design, Theoretical Model and Main Aim** | **Characteristics**  **of responders**  **and setting** | **Measures of behaviour**  Supplementary Information Table 1 | **Predictive measures of behaviour (Intention, plus other factors)** | **Predictive measures of Intention** | **Results** | **Main Outcomes** | **Quality** |
| --- | --- | --- | --- | --- | --- | --- | --- | --- |
| 1. Godin et al. (1998)  Canada **Understanding physicians intention to use a simple infection control measure** | Questionnaire Survey  TPB | GPs  Medical specialists Surgeons. | Measures of Behaviour  To use gloves whilst exposed to blood products or body fluids | Predictive Measures of Behaviour  **Intention**  ‘Do you intend to wear gloves when contact with blood or biological fluids is possible’  - Recorded as a percentage score | Predictive Measures of Intention  TPB variables:  -PBC Attitude  -Subjective Norm  -Perception of risk  -Gender  -Past Behaviour | **REPORTED BEHAVIOUR**  -Behaviour reported as intentional behaviour  -Intention to wear gloves when contact with blood or body fluids was strong 80%  **REPORTED PREDICTORS (Determinants) OF BEHAVIOUR**  - Intention to wear gloves was strong  **REPORTED PREDICTORS (Determinants) OF INTENTION**  1. S Norm*p<.0001  2.Attitude  3.PBC  **Additional Variables**  -Age; younger respondents had greater odds than older respondents | **SUBJECTIVE NORM main predictor of intention** | **Recruitment**  Random Sample  **Measurement**  Response Rate  40% (n=720) |
| 2. Levin (1999) USA **Test of the Fishbein and Ajzen models as predictors of health care workers glove use** | Questionnaire Survey  TRA, TPB  To establish predictors of **glove use** when there is potential exposure to blood | Nurses and laboratory workers | Measures of Behaviour  Glove use  -Correlation matrix for TRA and TPB variables | Predictive Measures of Behaviour  Intention measured not actual behaviour | Predictive Measures of Intention  TPB variables:  -PBC  -Intention  -Attitude  -Subjective Norm  **Plus:**  Perceived risk’ associated with glove us | **REPORTED BEHAVIOUR**  -Behaviour reported as intentional behaviour  -Only 30% of Health Care Workers thought that wearing gloves was completely under their control  **REPORTED PREDICTORS (Determinants) OF BEHAVIOUR**  -Not reported  **REPORTED PREDICTORS (Determinants) OF INTENTION**  Perceived control was the variable that contributed the most to the understandings of intentions toward glove use  Intention to wear gloves not influenced by important others (subjective norm) | **Main Outcome**  **ROLE OF PBC**  **ROLE OF ATTITUDE** (also important)  Professional Subjective Norm Differences not significant  **ROLE OF ADDITIONAL VARIABLES** (perceived risk: only measured in model) | **Recruitment**  -Random sample of nurses and laboratory workers  **Measurement**  -Elicitation Studies?  -Reliability?  **Psychometric quality and Response Rate**  - RR included 107 questionnaires were unusable who had no contact with blood during their working day |
| 3. Godin et al. (2000)  Canada **Determinants of nurses adherence to universal precautions for venipunctures** | Self-report  Survey  -Theory of Planned Behaviour  -Triandis’s Theory of Interpersonal Behaviour  -Aim to explain and predict adherence to Universal Precautions to performing venipunctures | 156 Registered Nurses working at a regional hospital  -92% women | Measures of Behaviour  -Adherence to Clinical Guidelines  -Use of gloves  -Hand washing  -Proper handling of the needle  -Use of puncture resistant containers for disposal and transportation of needles  Three month follow-up examined behaviour only:  -Nurses asked to estimate how many times they had adhered to UPs out of the last 10 VPs performed  Analysis | Predictive Measures of Behaviour  -Intention  -PBC  Plus:  -Perceived Barriers  -Habit  -Habit and Past Behaviour measured by asking respondents to estimate on a 5 point scale the proportion of time precautions | Predictive Measures of Intention  -Attitude  -Subjective Norm  -PBC  -Perceived normative belief  -Role belief  Assessed by means of several statements | **REPORTED BEHAVIOUR**  Rate of adherence to recommendations for 1248 hand hygiene recommendations was 70%  **REPORTED PREDICTORS (Determinants) OF BEHAVIOUR**  -Overall intention explained 0.68 of variance for measured behaviours  Strongest predictor of behaviour is intention (0.50)  -Perceived Barriers (0.44)  -PBC (0.43)  **REPORTED PREDICTORS (Determinants) OF INTENTION**  -Perceived barriers and personal normative beliefs highest correlation coefficients with intention (0.78 and 0.63)  -Personal normative belief (0.63) also significant  -All other variables (socio demographic) did not explain additional portions of variance of intention | **Main Outcome**  **ROLE OF ADDITIONAL VARIABLES**  -Perceived Barriers most powerful predictor of intention  **THE ROLE OF ADDITIONAL THEORETICAL VARIABLES**  -Triandis Theory : normative belief a significant contributor  -Limited effect of socio-demographic factors) | **Recruitment**  -Convenience Sample  -Conducted with a sample of limited record of needle stick injury  **Measurement**  **-**Response rate not reported  -Items for determinants of intention identified in the literature and not through elicitation studies  **Psychometric Quality and Response Rate**  -RR 72% (n=172)  Convenience sample  -Self-report measures, may have over-reported performance |
| 4. Watson & Myers (2001)  UK  **Which cognitive factors predict clinical glove use amongst nurses?** | Self Report Survey  -Theory of Planned Behaviour and TRA | 103 registered nurses employed at a teaching hospital  -25% of sample worked in A&Eand other general wards | Measures of Behaviour  Self-reported glove use behaviour | Predictive Measures of Behaviour  -Intention  -PBC  **Plus:**  -Perceived Barriers measured with 4 items on a 7 point scale | Predictive Measures of Intention  -Attitude  -Subjective Norm  -PBC | **REPORTED BEHAVIOUR**  - 35% said they would always wear gloves.  -43.7% strongly agreed they should wear gloves **REPORTED PREDICTORS (Determinants) OF BEHAVIOUR**  -The model explained 61% of actual glove use behaviour  -Self report glove use significantly correlated to PBC and intention  -Behaviour significantly **negatively** correlated with Barrier 1 ‘less likely to wear gloves if contact with blood is anticipated to be normal’  **REPORTED PREDICTORS (Determinants) OF INTENTION**  -Self-reported glove use significantly correlated with Attitude. Beta 0.632 p=<0.001  -PBC Beta 0.196 p=<0.05 | **Main Outcome**  **ROLE OF ATTITUDE**  (personal risk)  **ROLE OF ADDITIONAL VARIABLES**  **-**anticipated contact with blood also significant predictor of intention  **POWER OF INTENTION**  **-**Adding intentional variables only slightly (1%) increases explanatory power of the model | **Recruitment**  -Convenience Sample  **Measurement**  -Questionnaires delivered by hand by the Chief Nurse…and by hand by the investigators  -Self-report questionnaire  -Sig level for correlations set at < 0.01  **Psychometric Quality and Response Rate**  47.9% (n=103) |
| 5. O’Boyle et al. (2001)  USA  **Understanding adherence to hand hygiene recommendations: The Theory of planned behaviour** | -Self-report survey  -Non Participant observation  -Theory of planned behaviour  -Aim:  1. Estimate adherence to hand hygiene recommendations  2. Describe relationships among motivational factors  3. Test the TPB-based theoretical model to explain self-reported and observed hand washing | 120 Registered Nurses: 4 teaching hospitals in US. Intensive and Post Intensive care units | Measures of Behaviour  1. Adherence to hand hygiene recommendation (Self-report)  2.Observed Hand Hygiene  2-4 months later all participants were observed using the same protocol. | Predictive Measures of Behaviour  TPB variables:  -PBC  -Intention  **Plus:** Intensity of nursing activity  -Five indicators were used to construct an index reflecting intensity of activity in the nursing units | Predictive Measures of Intention  -Attitude  -Subjective Norm  Measures of Intention:  -HAI was also used to measure the motivational schema for hand washing | **REPORTED BEHAVIOUR**  **Actual Behaviour**  -after completion of care  -before giving care  -followed closely by removing gloves. These 3 indications accounted for 88% of observed indications  **REPORTED PREDICTORS (Determinants) OF BEHAVIOUR**  -Intention  -Observed Intensity of activity significantly negatively correlated to hand hygiene  **REPORTED PREDICTORS (Determinants) OF INTENTION**  -Control beliefs had a greatest effect on intention | **Main Outcome**  **ROLE OF PBC**  (The effect of PBC and its intermediate variable (Control Beliefs)  **POWER OF INTENTION**  Intention related to self-report NOT actual behaviour | **Recruitment**  Convenience Sample **Measurement**  -Previous observational studies (Larson et al. 1997) reported that inter-rater agreement for HOA scores was 100%  -Rooms with one to three patients  -Each room contained at least one sink. Sinks also available in halls and common work areas  -Relationship with investigator  **Psychometric Quality and Response rate**  21% (n=100)  -Interruption of observation of hand washing would affect the outcome of observation |
| 6. Bolman et al. (2002)  Holland **Factors determining cardiac nurses’ intentions to continue using a smoking cessation protocol** | Self-report  Survey  ASE Model  (Attitude, Social Influence and Self-efficacy) Aim:  First goal: To facilitate the continued use of a smoking cessation protocol  Second goal: to describe the applicability of ASE model; analyse the differences between intenders and non-intenders | Registered nurses in cardiology wards in 5 hospitals | Measures of Behaviour  -Use of smoking cessation protocol  Analysis  - t tests conducted to investigate differences in attitude, social influence and self-efficacy expectation beliefs between intenders and non-intenders  Analysis  Chi-square for gender, type of ward, training attended  Mann-Whitney U test for level of experience  Pearson correlations for ASE Concepts | Predictive Measures of Behaviour  ASE Model  **Plus:**  age, time worked on ward and number of hours per week, simplicity of guideline | Predictive Measures of Intention  -Attitude  -Social Influence  -Self-efficacy constructs | **REPORTED BEHAVIOUR**  -47% indicated they would probably go on to use protocol  -25% convinced they would go on to use  -20% unsure about continued use  **REPORTED PREDICTORS (Determinants) OF BEHAVIOUR**  - ASE Model explained 38% of intention to continue using the protocol  **PREDICTORS (Determinants) OF INTENTION**  -Only attitudinal subscales contributed to this explanation NOT social influence and self-efficacy  -Intention correlated most strongly with simplicity and perceived advantaged and self-efficacy expectations  -No difference in intentions found between smoking and non-smoking nurses | **Main Outcome**  **ROLE OF ADDITIONAL VARIABLES**  -Perceived Simplicity (part of ASE Model influencing attitude)  -No demographic difference between intenders and non-intenders  **SUBJECTIVE NORM DIFFERENECES**  Modelling and social support were not significantly associated with nurses intentions | **Recruitment**  Convenience Sample  **Measurement**  -No elicitation studies  -Small sample  -Results only reflect characteristics of responders  -Biased reports based on nurses expected to use protocol  -Nurses that had positive attitudes more likely to respond to survey than nurses who do not  **Psychometric Quality and Response Rate**  52% (n=85)  -Elicitation studies not performed but scales developed based on previous literature |
| 7. Jenner and Watson et al. (2002)  **Explaining hand hygiene practice: An extended application of the Theory of Planned Behaviour** | Cross-sectional questionnaire  TPB and constructs from Health Belief Model  Develop a theoretical framework to identify perceived cognitive and physical factors (variables) that may explain hand hygiene behaviour | Nurses (n=76)  Therapists (n=17)  Health Care Assistants (n=4) | Measures of Behaviour  Target Behaviour of hand washing defined in accordance to national guidelines.  Analysis  -Non-parametric bivariate correlations for relationship between predictive constructs and dependant variables (intention and behaviour) | Predictive Measures of Behaviour  TPB variables:  -PBC  -Intention  -Attitude  -Subjective Norm  **Plus:**  -Personal Responsibility  -Barriers: time,  availabilityand number and location of sinks | Predictive Measures of Intention  TPB variables  -Attitude  -PBC  -SNorm | **REPORTED BEHAVIOUR**  -Intention was a strong predictor of behaviour with 79% of cases correctly classified  -PBC and intention significant predictors of behaviour  -personal responsibility significantly correlates with behaviour  -Barriers contributed an additional 10%  **REPORTED PREDICTORS (Determinants) OF BEHAVIOUR**  -Not reported  **REPORTED PREDICTORS (Determinants) OF INTENTION**  -Attitudes and personal responsibility significant predictors of intention  -The model correctly classified 79% cases of intention to perform appropriate hand washingand 87% of hand hygiene behaviour | **Main Outcome**  **ROLE OF ADDITIONAL**  **THEORETICAL VARIABLES**  (Dominant Predictor - effect of personal responsibility)  **ROLE OF ATTITUDE**  (The effect of attitudes on intention)  **POWER OF INTENTION** (adding variables) | **Recruitment**  Convenience Sample  **Measurement**  -Self report measures could be a over-estimation of intention and behaviour  -Difficulty in recruiting other professional groups  -No elicitation studies  **Psychometric Quality and Response Rate**  34% (n=97) |
| 8. Limbert and Lamb (2002)  **UK Doctors use of clinical guidelines: Two applications of the theory of planned behaviour** | Questionnaire Survey:  and interviews TPB  Intentions to use Clinical Guidelines | Study 1: 15 randomly selected Doctors (interviews)  Study 2: 346 Surgeons and Physicians below level of Consultant | Measures of Behaviour  Guideline use by hospital doctors  -Guideline for the management of acute asthma in an A&E Department  -Plus antibiotic guideline use by senior doctors | Predictive Measures of Behaviour  TPB Variables: Intention, PBC  **Plus:** For Antibiotic questionnaire:  -individuality  -evidence  -Usefulness | Predictive Measures of Intention  TPB Variables: Attitude, PBC, SNorm | **REPORTED BEHAVIOUR**  Asthma Guideline  Respondents reported using the guideline treating Asthma patients  Antibiotic Guideline  -Attitudes towards the guideline were positive  **REPORTED PREDICTORS (Determinants) OF BEHAVIOUR**  -Not reported  **REPORTED PREDICTORS (Determinants) OF INTENTION**  -Asthma Guideline: TPB explained 58% of variance in intention  -SNorm the most powerful predictor of intention  -Attitude was the strongest predictor of intention to use antibiotic guideline  - Perceived Usefulness also strongly correlated with intention | **Main Outcome**  **SUBJECTIVE NORM DIFFERENCES**  (Asthma Guideline) Professional status influences intentional behaviour)  **ROLE OF ATTITUDE**  (role of beliefs underpinning attitude: antibiotic guideline)  **ROLE OF ADDITIONAL VARIABLES** (perceived usefulness) | **Recruitment**  First Study: Random sample  Second study: Convenience sample  **Measurement**  Elicitation studies for original questionnaire  -Results of these studies may be confounded by differences between guidelines used in each study  **Psychometric Quality and Response Rate**  Questionnaire 1: 78% (n=223)  Questionnaire 2: 62% (n=214)  -Informed by unstructured elicitation studies |
| 9. Beatty and Beatty (2004) **Anaesthetists intentions to violate safety guidelines** | Questionnaire Survey  TPB  Aim: Investigate likelihood anaesthetists will violate safety guidelines on**:**  -Visiting patients before surgery  -Performing pre-anaesthetic equipment checks  -Silencing of alarms during anaesthesia | Anaesthetists  -Most common grade id consultants | Measures of Behaviour  Three violations to safety:  1. Failing to visit patients before surgery  2.Failure to perform pre-anaesthetic equipment checks  3.Silencing of alarms during anaesthesia  Analysis  -Mean scores on main behaviours  -Pearson R on reported behaviour and intentional (and additional variables) | Predictive Measures of Behaviour  TPB variables:  -Intention  **Plus**:  Personal Norms which includes:  -moral norms  -anticipated regret  -personal identity  -habit | Predictive Measures of Intention  -PBC  -Attitude  -Subjective Norm | **REPORTED BEHAVIOUR**  -Routine violations common (indicated by personal norm scores)  **REPORTED PREDICTOR (Determinants) OF BEHAVIOUR**  -Not reported  **REPORTED PREDICTOR (Determinants) OF INTENTION**  -All correlations to TPB variables quite low apart from normative belief  -Normative beliefs (the opinion of significant others would hold about them performing the violation)  -Personal norms and Habit  -The more intense the anaesthetists beliefs that the violations were important, the less likely they were to violate -except for the case of alarm silencing | **SUBJECTIVE NORM DIFFERENCES**  -Role of Normative Belief  **ROLE OF ADDITIONAL THEORETICAL VARAIBLES**  -Personal Norms  - Habit dominant coefficient across all violations  **ROLE OF ATTITUDE** (intensity of beliefs) | **Recruitment**  Convenience sample **Measurement**  -In questionnaire-based studies of this sort…response rates of 30% are normally considered good.  **Psychometric Quality and Response Rate**  42.7% (N=114)  -bias sample, self-selecting |
| 10. Maue and Segal et al. (2004)  USA  **Predicting physician guideline compliance: An assessment of motivators and perceived barriers** | TPB and TRA  Questionnaire Survey  Aim  Observe practitioners compliance with practice guidelines | All Practitioners: staff physicians, physician residents, interns, advanced nurse practitioners and physician assistants | Measures of Behaviour  A variety of Guidelines. Although intentional measures not specifically related to each individual guideline.  Guidelines  -24 providers implemented Dyspepsia guidelines  -Oncology and haematology service implemented guidelines  - COPD guidelines  asthma guidelines | Predictive Measures of Behaviour  TRA and TPB adapted for this study variables:  -PBC  -Intention  -Attitude  -Subjective Norm  **Plus:**  - Personal control  -Internal barriers: confidence, understanding and practice habits.  External barriers: patient demands, time constraintsand delays in receiving lab results | Predictive Measures of Intention  Attitude  -Subjective Norm  For each antecedent the lower the score the more positive the attitude, intention, subjective norm | **REPORTED BEHAVIOUR**  The mean self-report compliance behaviour was 65%, compliance assessed by chart review was 54%  **REPORTED PREDICTORS (Determinants) OF BEHAVIOUR**  SNorm a significant determinant (social pressure) indicated a motivation to comply with guideline  PBC also significant  -Past behaviour: similar to current behaviour  -External Barriers more inhibiting than internal barriers  **REPORTED PREDICTORS (Determinants) OF INTENTION**  Positive correlations between: attitude, SNorm, PBC, internal barriers, | **Main Outcome**  **ROLE OF PBC (**r=0.73) Most significant  -Internal barriers most significant in PBC  **PROFESSIONAL SUBJECTIVE NORM DIFFERENCES**  **ROLE OF NON-THEORETICAL ADDITIONAL VARIABLES**  (Organisational factors as external barriers inhibitor r= -0.50, p < .05)  **MEASUREMENT OF INTENTION**  (intention and self-report not significant (r=0.13) | **Recruitment**  Convenience Samples Survey 1 administered immediately preceding guidelines introduction to measure predictors of guideline compliance  Survey 2: Administered 4 months after implementation of guidelines  **Measurement**  -Problem with self-report: biased by response social desirability, acquiescence  -Not a random sample  **Response Rate**  Survey 1 63% (n=106)  Survey 2: 51% (n=36) |
| 11. Puffer and Rashidian (2004) UK  **Practice nurses intentions to use clinical guidelines** | Questionnaire Survey:  TPB  Examine Utility of TPB in explaining variations in practice nurses intentions to provide smoking cessation advice according to CHD Guidelines | **Registered Practice Nurses** (all female) | Measures of Behaviour  Elicitation studies carried out: semi-structured interviews | **Behaviour**  -Not Measured  **Intention**  TPB variables:  -PBC  -Intention  -Attitude  -Subjective Norm  Plus:  -Past Behaviour | Measures of Intention  **Dependant variable**: behavioural intention, measured with 2 items | **REPORTED BEHAVIOUR**  Smoking cessation advice  **REPORTED PREDICTORS (Determinants) OF BEHAVIOUR**  -Only predictors of intention measured  **REPORTED PREDICTORS (Determinants) OF INTENTION**  -PBC had the strongest relationship with intention (r=0.546)  -Attitude (r=0.450)  -Past Behaviour (r=0.382)  -Indirect PBC (r=0.306)  -Indirect attitude (r=0.306)  -indirect SNorm (r=0.300  -Indirect measures of attitude and PBC explained | **Main Outcome**  **ROLE OF PBC**  (Ability to carry out behaviour strongest correlation with intention r=0.546)  **ROLE OF NON-THEORETICAL ADDITIONAL VARIABLES**  (Inclusion of past behaviour, ageand work characteristics did not influence behaviour: but not reported)  -Belief items indicate lack of time and training | **Recruitment**  Convenience Sample  **Measurement**  -Elicitation studies (semi-structured interviews)  -Reliability of items tested  -Only one item of Subjective Norm measured  -Limited sample  **Response Rate**  RR 54% (n=48) |
| 12. Foy and Walker et al. (2005)  UK  **Theory-based identification of barriers to quality improvement: induced abortion care** | Questionnaire Survey  Based on the TPB  Elicitation studies based on previous lit review and case studies  Based on: INDUCED ABORTION CARE  1. Offer of an assessment appointment within 5 days  2.Supply of contraceptives at discharge | -26 hospitals in Scotland  - **All clinical staff i**n 26 Gynaecology units  -Part of a cluster randomised controlled trial  -The study population comprised all clinical staff involved in abortion care at the 13 units randomised to the intervention arm | Measures of Behaviour  Compliance with the guideline ‘The Care of women requesting induced abortion’ | Predictive Measures of Behaviour  TPB variables:  -PBC (five items)  -Intention  -Attitude  -Subjective Norm  **Plus:**  -Specific facilitators and barriers (brief open-ended questions) | Predictive Measures of Intention  -Attitude (four items)  -Subjective Norm (three bipolar items) | **REPORTED BEHAVIOUR**  -Offer of assessment 45.8% (median compliance)  -To supply contraceptive at discharge. 58.6%  -Mean intentions to comply with both behaviours were high.  **REPORTED PREDICTORS (Determinants) OF BEHAVIOUR**  -Intention and PBC best explained compliance to the 2 behaviours  -Mean perceived PBC was lower for the offer of assessment appointment  - PBC was added to intention – predicting 15% of variation in unit compliance  **REPORTED PREDICTOR (Determinants) OF INTENTION**  ‘Subjective Norm’ best predicted intention | **Main Outcome**  **PROFESSIONAL SUBJECTIVE NORM DIFFERENCES**  (Subjective norm most influential on assessment appointment)  **ROLE OF PBC** (on supply of contraceptives, but r 0.152 does not look significant see table 5)  -Organisational constraints for PBC –in this instance cost  **POWER OF INTENTION**  (increased when PBC was added to intention predicting 15% of variation in unit compliance) | **Recruitment**  Random sample  **Measurement**  -Elicitation studies  -Pilot tested questionnaire  -Measures of psychological variables all achieved  -Cronbach’s Alpha acceptable reliability  **Response Rate**  Response to survey: 74% (n=151) from the 12 units that participated in the survey |
| 13. Pessoa-Silva and Posay-Barber et al. (2005)  Switzerland  **Attitudes and perceptions toward hand hygiene among healthcare workers caring for critically ill neonates** | Questionnaire Survey  Based on TPB  To identify beliefs and perceptions associated with intention to comply with hand hygiene | Health Care Workers  49 Nurses  12 Physicians University of Geneva Hospitals  -Conducted in one neo-natal unit  -Hand hygiene facilities conveniently located throughout the unit | Measures of Behaviour  Intention to comply with hand hygiene was the Dependant Variable | Predictive Measures of Behaviour  -Intention  -Perception of risk of transmission  -Motivation: ‘Do you feel you can improve your compliance with hand hygiene’ | Predictive Measures of Intention  -Attitude  -Perceived difficulty  -Perceived SNorm  -Perceived behavioural norm  -Perception of risk | **REPORTED BEHAVIOUR**  -Behaviour reported as intentional behaviour  **REPORTED PREDICTORS (Determinants) OF BEHAVIOUR**  -A positive intention to comply with hand hygiene was found amongst 64% of respondents  -Rate of intention as low as 18%  -Of the 49 nurses and 12 physicians responding 75% believed that they could improve their compliance with hand hygiene  **REPORTED PREDICTOR (Determinants) OF INTENTION**  -Intention to comply associated with the perceived control  -And a positive perception of how superiors valued hand-hygiene  -A positive attitude also influential | **Main Outcome**  **ROLE OF PBC** (Intention to comply perceived control over the difficulty to perform hand hygiene – reported as odds ratio 4.01)  **PROFESSIONAL SUBJECTIVE NORM DIFFERENCES**  (Influence of professional colleagues )  **ROLE OF ATTITUDE**  Attitude also an influence | **Recruitment**  Convenience sample  **Measurement**  -Infection control staff distributed the questionnaire – did this reinforce social desirability bias?  -Social and environmental pressure may account for behaviour rather than intention  **Response Rate**  76% (n=61) |
| 14. Bonnetti and Johnston et al. (2010)  UK  **Applying psychological theories to evidence-based clinical practice: identifying factors predictive of placing preventative fissure sealants** | Questionnaire Survey  Design was a predictive study with theoretical variables and outcomes.  -TPB  - Social Cognitive Theory  -Common Sense Self-regulation Model  -Operant Learning Theory  Implementation Intention, Stage model and knowledge (a non-theoretical model)  This study explored the usefulness of a range of models to predict placing of fissure sealants | General Registered Dental Practitioners. Target sample size of 200 based on recommendation (Green 1991) to have a minimum of 162 when undertaking multiple regression analysis with 14 predictor variables  -GDP 58% male. Sample | Measures of Behaviour  Two behaviours:  -**Behavioural Simulation** (scenario decision-making) – three elements identified by SIGN guideline and , expert opinion  -**Behavioural intention:**  -Assessed by three items | Predictive Measures of Behaviour  Constructs that predicted behavioural simulation were:  -Scenario decision-making  -Behaviour intention | Predictive Measures of Intention  -More evidence-based behaviour may be achieved by influencing beliefs about the positive outcomes and building a habit as part of placing them!! | **REPORTED BEHAVIOUR Behavioural Simulation**  -Intention Beta 0.48 p<.01  **REPORTED PREDICTORS (Determinants) OF BEHAVIOUR**  Behavioural simulation predictors:  -TPB 31%  -SCT 29%  -OLT 30%  -II 7%  -Habit (OLT), timeline acute (CS-SRM) and outcome expectancy (SCT) entered the equation together explaining 38% of variance  -Common Sense Model did not explain any variance in intention  **REPORTED PREDICTOR (Determinants) OF INTENTION**  -Habit 0.35 Beta p=0.001  -Attitude 0.25 Beta | **Main Outcome**  **ADDITIONAL THEORETICAL VARIABLES**  (Habit as a predictor of INTENTION)  **ROLE OF ATTITUDE**  (Direct and indirect Normative Belief)  **POWER OF INTENTION**  (Power of various models testedand VARIABLES IN MODELS) | **Recruitment**  Random Sample: **Scottish general practice** board list by a statistician using a list of random sampling numbers  **Measurement**  -Elicitation studies semi-structured interviews  -Postal reminders sent at 2, 4 and 6 weeks  - internal consistency of items measured by Cronbach’s alpha if <6.0 then item removed  **Response Rate**  29% (n=120/407) |
| 15. Kortteisto et al. (2010) Finland  **Healthcare professionals’ intentions to use clinical guidelines: a survey using the theory of planned behaviour** | Questionnaire Internet Survey  -TPB  -Finish Clinical Guidelines evolving to be in electronic health record.  Discover the general level of guideline use :  Do healthcare pro’s have a negative or positive intention toward guideline use?  -Do and how do healthcare pro’s differ in their intentions? | -Nurses, physiciansand other HCPs  -Finnish healthcare organisations, within three hospital districts  Units of Dental Care, radiology, laboratory workers were excluded | Measures of Behaviour  Clinical Practice Guideline use in general  -The target behaviour is considered to involve a professionals knowing use of patient specific guidelines in clinical decision making | Predictive Measures of Behaviour  Behaviour:  Behavioural intention:  -The Dependent Variable was intention  -Profession and Organisational characteristics also considered:  -Individual variables  -Gender  -Age  -Primary Care | Predictive Measures of Intention  TPB variables:  -PBC  -Attitude  -Subjective Norm  -Attitude measured by 3 behavioural beliefs  -Subjective Norm assessed by three normative beliefs about social pressures to use clinical guidelines  -PBC assessed with 6 controlled beliefs about context | **REPORTED BEHAVIOUR**  Intention to use clinical guidelines in practice more often positive than negative  **REPORTED PREDICTORS (Determinants) OF BEHAVIOUR**  -Overall 18% indicated absolutely positive intention  -30% positive intention  -1% indicated absolutely negative  -4% negative views  **REPORTED PREDICTORS (Determinants) OF INTENTION**  -Overall regression model explained only 36% of intention  -Physicians main determinant PBC  -Nursesand other Health Care Pros the main determinant was SNorm | **Main Outcome**  **POWER OF INTENTION**  (TPB did not capture a large proportion of intention)  **ROLE OF PBC**  (a significant predictor of be intention for physicians)  **PROFESSIONAL SUBJECTIVE NORM DIFFERENCES**  (nurses subjective norm a significant predictor of intention) | **Recruitment**  Convenience Sample  **Measurement**  -RR attempted to be improved: email invitation followed up by two reminders  -Pilot Questionnaire increased response rate  -Purely questionnaire-based, no observation  -Does not take into account effects of additional variables  -Limited explanation of intentions as a stand alone model  -Cultural differences in intention (Finnish)  **Response Rate**  RR 36% (n=806) |
| 16 Leitlen *et al.* (2011) Factors influencing Dutch practice nurses’ intention to adopt a new smoking cessation intervention | Questionnaire Survey  The I-Change Model  Establish differences in intentions and determinates of intention between adopters and non-adopters | Practice Nurses and Nurse Practitioners working in General Practice | Measures of Behaviour  Adopting a new smoking intervention | Predictive Measures of Behaviour  Not measured. Intention was only predictor of behaviour | Predictive Measures of Intention  -Attitude  -Social Influence  -Self-efficacy | **REPORTED BEHAVIOUR**  The majority of practice nurses did not intend to adopt the intervention  **REPORTED PREDICTORS (Determinants) OF BEHAVIOUR**  Not measured  **REPORTED PREDICTORS (Determinants) OF INTENTION**  Adopters  -Positive attitude  -Perceived social influence (social norms and support)  -No differences in differences in demographics | **Main Outcome**  **Intention to adopt influenced by:**  **Attitude**  Statistically significant p=<.05  Social Norms p=<.05 | **Recruitment**  Self-selecting  Measurement  **Role differences: A potential mediator**  **Response Rate**  Not reported  ->20% of questionnaires not appropriately completed |
| 17 Buenestado *et al.* (2013) Evaluating acceptance and user experience of a guideline-based clinical decision support system execution platform | Questionnaire Survey  Technology Acceptance Model  Aim: Understand intention of Paediatrician Physicians towards prolonged (3 month) use of a Computerised Asthma Clinical Guidelines for diagnosis and treatment  Is intention modified by continued use | Spain  Basque Health care services | Measures of Behaviour  Two CCGPs, one for the diagnosis and initial treatmentand the other on continuation of treatment | Predictive Measures of Behaviour  Intention  Habit | Predictive Measures of Intention  Determinants identified in Technology Acceptance Model:  -Perceived Usefulness (PU)  -Perceived Ease of Use (PEU)  -Compatibility  -Attitude  -SNorm | **REPORTED BEHAVIOUR**  -Initial disposition to use e-guides is good  **REPORTED PREDICTORS (Determinants) OF BEHAVIOUR**  -Lower values in compatibility and habit indicate difficulty in integrating into daily routine  **REPORTED PREDICTORS (Determinants) OF INTENTION**  ‘Facilitators’ the variable most strongly associated with intention | **Main Outcome**  Intention measured after a few weeks  **Facilitators:**  -Perceived Usefulness  -Attitude  -SNorm | **Recruitment**  Convenience Sample  **Measurement**  **-No elicitation study**  **-Questionnaires for 8 physicians**  **Response Rate**  **-8 volunteers** |
| 18. Perez *et al.* (2014) TPB can help to understand processes underlying the use of two emergency medicine diagnostic imaging rules | Questionnaires Survey  TPB  Aim: Comparing intentions from 2 disgnostic imaging rules in the Emergency Department | Canada  Physicians in Emergency Department | Measures of Behaviour  Use of  -C Spine Rule (in one hospital)  -CT Head Rule (in one hospital) | Predictive Measures of Behaviour  Intention | Predictive Measures of Intention  TPB predictive variables  -Attitude  -SNorm  -PBC | **REPORTED BEHAVIOUR**  -Intention only associated with actual behaviour in one hospital (for CCR) not for CDR  **REPORTED PREDICTORS (Determinants) OF BEHAVIOUR**  -Not reported  **REPORTED PREDICTORS (Determinants) OF INTENTION**  -Attitudesand subjective norms predictive of intention in both hospitals | **Main Outcome**  **-Intention significantly associated with actual behaviour for CCR**  **-Constructs outside TPB should be considered to understand CRDS** | **Recruitment**  -Randomised sample  **Measurement**  -No elicitation study  **Response Rate**  **-** 223 of the 378 eligible physicians completed their assigned baseline  survey |

Exploring Health Professionals Instrumental RU Intentions

Attitude and personal

risk

PBC and Subjective Norm as theoretical predictors

i

Intention

Intention as a predictor of behaviour

i

Intention

Increasing the predictive value of intention

i

Intention

The role of additional

variables

Professional subjective norm differences

The role of PBC (varying effects and professional differences)

Influence of attitude

on intention

**File 2:** Preliminary Analysis: Identifying Main Outcomes

*Interpretation (inductive reasoning)*

*Interpretation (inductive reasoning)*

*Interpretation (inductive reasoning)*

Competing explanations for the prediction of intention and intention on behaviour

Differences and similarities in how health professional groups form intentions

**File 3**: Grouping Main Outcomes

Theoretically-based variables as dominant predictors of intention and intention of behaviour

-The role of additional variables

-Increasing the predictive value of Intention

-Professional Subjective Norm Differences

-Varying Professional effects of Perceived Behavioural Control (PBC)

-Influence of attitude on intention

-Attitude and Personal risk

-Intention as a predictor of behaviour

-PBC and Subjective Norm as theoretical predictors

**Supplementary Information Table 2: Theoretically based variables as dominant predictors of intention (Group 1)**

| **Predictor Variable** | **Source** | **Intention Model** | **Health Professional Group** | **Behaviour** | **Predictor and Statistical Significance** |
| --- | --- | --- | --- | --- | --- |
| **Attitude or Behavioural Belief** | Watson & Myers (2001)  Limbert and Lamb (2002)  Bonetti *et al.* (2010)  Leitlen  *et al.* (2011)  Perez *et al.* (2014) | TRA and TPB  TPB  TPB  I-Change  TPB | Nurses  Surgeons and Physicians  Dental Practitioners  Practice Nurses, Nurse Practitioners  Physicians | Glove use (when potential for blood exposure)  Prescribing Antibiotics Guideline  Placing preventative fissure sealants  Smoking Cessation Intervention  C Spine and CT Head Rules | r= 0.63 (attitude)  r= 0.86 p= < 0 .001 (attitude)  Beta 0.29 p=0.01 (behavioural belief)  t= -7.36 p=<0.001 (attitude)  beta=0.4 p=<0.001 (attitude) |
| **Subjective Norm or Normative Belief** | Godin *et al.* (1998)  Limbert and Lamb (2002)  Beatty and Beatty (2004)  Foy *et al.* (2005)  Kortteisto *et al.* (2010)  Leitlen  *et al.* (2011)  Perez *et al.* (2014) | TPB  TPB  TPB  TPB  TPB    I-Change  TPB | Physicians  Junior Doctors  Anaesthetists  All Clinical Staff involved abortion care  Nurses, other Health Care Professionals  Practice Nurses, Nurse Practitioners  Physicians | Glove use (contact with body fluids)  Acute Asthma Guideline  Anaesthetists Safety Guidelines (pre-op visits)  Offer of assessment appointment induced abortion  General Guideline use  Smoking Cessation Guidelines  C Spine and CT Head Rules | Odds Ratio 14.61  p=<.0001 (subjective norm)  r=0.71 p= < 0 .001 (subjective norm)  Mean 67.9% (normative belief)  r=0.52 p=<0.01 (subjective norm)  beta= 0.33 p = <0.001 (subjective norm)  t=-0.71 p=<0.001  r=0.26 p=<0.001 |
| **PBC or Control Belief** | Levin (1999)  O’Boyle *et al.* (2001)  Puffer and Rashidian (2004)  Pessoa-Silva *et al.* (2005)  Foy and Walker (2005)  Kortteisto *et al.* (2010)  Buenestado *et al.* (2013) | TRA, TPB  TPB  TPB  TPB  TPB  TPB  TAM | Nurses and Laboratory workers  Nurses  Nurses  Nurses and Physicians  All Clinical Staff involved abortion care  Physicians  Physicians | Glove Use (when potential for blood exposure)  Adherence Hand Hygiene  Smoking cessation (CHD Guidelines)  Hand Hygiene with Neonates  Offering contraceptive supplies at discharge  General Guideline use  Computerised Asthma Guidelines | R2 0.78 p = < .01 (PBC)  r= 0.557 p < 0.05 (Control Beliefs)  r= 0.546 p= < 0.001 (PBC)  Odds Ratio 4.01 p = < .01  (PBC)  r= 0.15 (PBC)  beta 0.45 p = <0.001  r=0.89 |

**Supplementary Information Table 3: Studies which measured intention and the association with behaviour (Group 1)**

| **Intention** | Levin (1999)  Watson and Myers (2001)  Godin *et al.* (2000)  O’Boyle *et al.* (2001)  Limbert and Lamb (2002)  Jenner *et al.* (2002)  Maue *et al.* (2004)  Bonetti *et al.* (2010)  Perez *et al.* (2014) | TRA and TPB  TPB  TPB  TPB  TPB  TPB  TRA and TPB  TPB  TPB | Nurses and Laboratory Workers  Nurses  Physicians  Nurses  Junior Doctors and senior registrars  Nurses and Health Care Assistants  Physicians Advanced Registered Nurse Practitioners  Dental Practitioners  Physicians | Glove Use. (Potential for blood exposure)  Glove Use. (Potential for blood exposure  Universal precautions for venipunctures  Adherence Hand Hygiene (Self-report and observed)  Asthma (Junior Doctors) Antibiotic (senior Doctors)  Adherence to Hand Hygiene Guidelines  General Guideline use  Physicians and  (Placing Fissure Sealants)  C Spine Rule  CT Head Rule | Behaviour (self-report)  Behaviour (self-report)  Behaviour (self-report)  Behaviour (self-report)  Observed Behaviour  Behaviour (self-report)  Behaviour (self-report)  Behaviour (self-report)  Behaviour (Simulated)  Behaviour (self-report) | r=0.47 p=<0.01 (not wear in past month)  r=0.69 p= < 0.01  r=0.50 p=<.0001 (how many times adhered to UP Guidelines)  r =0.38 p= < .001  r=0.68 (not sig)  Asthma r=0.40 p=<.0001  Antibiotic r=0.31 p=<.0001  Beta 4.53 p=<0.001  R= 0.13  r= 0.50 p= < 0.01  OR 1.79 p= < 0.01  OR 1.05 p= 0.60 |
| --- | --- | --- | --- | --- | --- | --- |

**Supplementary Information table 4: Differences and similarities in how Health Professionals form intentions (by behaviour) (Group 2)**

| **Behaviour** | **Professional Group** | **Main variables predictive of**  **Intention** |
| --- | --- | --- |
| **Glove Use**  Godin *et al.* (1998)  Levin (1999)  Watson & Myers (2001) | Physicians  Nurses and Laboratory  Workers  Nurses | Subjective Norm Odds Ratio  14.61 p= <.0001  PBC R= 0.29 p = < 0.05  Attitude R2 0.63 p = < .01 |
| **Hand Hygiene**  O’Boyle *et al.* (2001)  Jenner *et al.* (2002)  Pessoa-Silva *et al.* (2005) | Critical Care Nurses  Nurses, Therapists and HCAs  Nurses and Physicians | Control Beliefs R= 0.557 p < 0.05  Personal Responsibility R= 0.42 p < 0.01  PBC Odds Ratio 4.01 p = < .01 |
| **General Guideline Use**  Kortteisto *et al.* (2010)  Maue *et al.* (2004) | Physicians, Nurses and Other Professionals  Physicians and Advanced Registered Nurse Practitioners | PBC (Physicians) beta 0.45 p = <0.001  Subjective Norm (Nurses) p = <0.01  Subjective Norm (other professionals) p = <0.01  Internal barriers (confidence, understanding, practice habits) r = -0.73, P <0.0001 **(Behaviour)** |
| **Specialist Guideline Use**  Universal precautions for venipunctures (Godin *et al.* 2000)  Smoking Cessation (Bolman *et al.* 2002)  Smoking Cessation (Leitlen  *et al.* 2011)  Acute Asthma and Antibiotic Guideline (Limbert and Lamb 2002)  Anaesthetists Safety Guidelines (Beatty and Beatty 2004)  CHD Guidelines and Smoking Cessation advice (Puffer and Rashidian 2004)  Assessment and care of induced abortion (Foy *et al.* 2005)  Placing preventative fissure sealants (Bonnetti *et al.* 2010)  **Clinical Decision Support Aids**  Buenestado *et al.* (2013) Paediatricians use of decision-aid  Perez *et al.* (2014) Use of clinical decision rules | Physicians  Nurses  Practice Nurses and Nurse Practitioners  Junior Doctors (Asthma)  Surgeons and Physicians below level of consultant (Antibiotic Guideline)  Anaesthetists  Practice Nurses  All Clinical Staff involved abortion care  Dental Practitioners  Physicians  Physicians | PBC – Control Beliefs, perceived Barriers R2 0.62 p < .001  Perceived Simplicity r = 0.65 p < 0.01  Attitude t=-7.36 p=<0.001  Subjective Norm t=-0.71 p=<0.001  Subjective Norm (Asthma) R=0.71  p= < 0 .001  Attitude (Antibiotics) r=0.61 p= < 0 .001  Attitude (usefulness of evidence) r = 0.86  Normative Belief (mean scores)  - No pre-op visit = 67.9 %  - No cockpit checks = 67.1 %  - Silence alarms = 38.3%  Habit (no pre-op visit) r=0.62 p=< 0 .01  Habit (no cockpit checks) r=0.79 p=< 0 .01  Habit (silence alarms) r=0.47 p=< 0.01  **(Behaviour)**  PBC r= 0.546 p= < 0.001  Subjective Norm R=0.52 p=<0.01 (Offer assessment appointment )  PBC R= 0.15 p not reported (offering contraceptive supplies at discharge)  Habit R= 0.75 p = <0.001 **(Behaviour)**  Perceived Usefulness (PU) r =0.84  Perceived Ease of Use (PEU) r=0.87  Attitude r=0.88  Attitude r=0.4 p=<0.001  Subjective Norms r=0.26 p=<0.001 |

**Supplementary Information Table 5: Differences in how professional groups form intentions (Group 2)**

| **Professional Group** | **Source** | **Behaviour** | **Main Predictor of Intention** |
| --- | --- | --- | --- |
| **Nurses** | Levin (1999)  Watson & Myers (2001)  Boyle et al. (2001)  Jenner et al. (2002)  Pessoa-Silva and Posfay-Barbe et al. (2005)  Kortteisto et al. (2010)  Maue et al. (2004)  Bolman et al. (2002)  Puffer and Rashidian (2004)  Foy and Walker (2005)  Leitlen *et al.* (2011) | Glove use when potential for blood exposure  Glove use when potential for blood exposure  Hand Hygiene  Hand Hygiene  Hand Hygiene  General Guideline Use  General Guideline Use  Smoking Cessation Guidelines  CHD Guidelines and Smoking Cessation advice  Abortion Care  Smoking Cessation | PBC R= 0.29 p = < 0.05  Attitude R2 0.63 p = < .01  Control Beliefs R= 0.557 p < 0.05  Personal Responsibility R= 0.42 p < 0.01  PBC Odds Ratio 4.01 p = < .01  Subjective Norm (Nurses) p = <0.01  PBC (internal barriers) r = -0.73, P <0.0001  Simplicity r = 0.65 p < 0.01  PBC r= 0.546 p= < 0.001  Subjective Norm R=0.52 p=<0.01 (Offer of assessment appointment )  PBC R= 0.15 p not reported (Offering contraceptive supplies at discharge)  Attitude t=-7.36 p=<0.001  Subjective Norm t=-0.71 p=<0.001 |
| **Physicians** | Godin *et al.* (1998)  Pessoa-Silva *et al.* (2005)  Kortteisto *et al.* (2010)  Maue and Segal *et al.* (2004)  Godin *et al.* (2000)  Limbert and Lamb (2002)  Beatty and Beatty (2004)  Foy and Walker (2005)  Buenestado *et al.* (2013)  Perez *et al.* (2014) | Glove use when potential for body fluid or blood exposure  Hand Hygiene  General Guideline Use  General Guideline Use  Universal precautions for venipunctures  Asthma Guideline (Junior Doctors)  Antibiotic Guideline (Surgeons and Physicians)  Anesthetists Safety Guidelines  Abortion Care  Clinical Decision Support Aids  Decision Rules | Subjective Norm Odds Ratio  14.61 p=<.0001  PBC Odds Ratio 4.01 p = < .01  Subjective Norm (Nurses) p = <0.01  PBC (internal barriers)r = -0.73, P <0.0001  Perceived Barriers R2 0.62 p < .001 (to be performed in next three months)  Subjective Norm R=0.71 p= < 0 .001  Attitude (usefulness of evidence) r = 0.86  Personal Norm Habit (no pre-op visit) r=0.62 p=< 0 .01  Personal Norm Habit (no cockpit checks) r=0.79 p=< 0 .01  Personal Norm Habit (silence alarms) r=0.47 p=< 0.01  Subjective Norm R=0.52 p=<0.01 (Offer of assessment appointment )  PBC R= 0.15 p not reported (Offering contraceptive supplies at discharge)  Perceived Usefulness (PU) r =0.84  Perceived Ease of Use (PEU) r=0.87  Attitude r=0.88  All p=<.05  Attitude r=0.4 p=<0.001  Subjective Norms r=0.26 p=<0.001 |
| **Other Health Care**  **Groups**  Dental Practitioners  Laboratory Workers  Other Professionals | Bonnetti *et al.* (2010)  Levin (1999)  Kortteisto *et al.* (2010)  Foy *et al.* (2005) | Placing preventative fissure sealants  Glove use when potential for blood exposure  General Guideline Use  Abortion Care | Habit R= 0.75 p = <0.001  PBC R2 0.78 p = < .01  Subjective Norm (Nurses) p = <0.01  Subjective Norm R=0.52 p=<0.01 (offer of assessment appointment)  PBC R= 0.15 (Offering contraceptive supplies at discharge) |

**Supplementary Information table 6: Competing explanations for the prediction of intention and behaviour (Group 3)**

| **Behaviour** | **Professional**  **Group** | **Additional Variable**  **(extension of model)** | **Association to Intention** | **Association to Behaviour** | **Theoretical**  **Base** |
| --- | --- | --- | --- | --- | --- |
| **Glove Use**  Godin *et al.* (1998)  Levin (1999)  Watson and Myers (2001) | Physicians  Nurses and Lab Workers    Nurses | Habit of wearing gloves  Perceived risk of infection  Age Group (year)  20-39  Comparative Risk  Perceived Barrier:  If volume of blood is minimal  If short of time | Odds Ratio 3.01 p=<.0032  Odds Ratio 2.77 p=<.0030  Odds Ratio 2.77 p=<.0041  r=.07 not significant  NR    NR | NR    NR  r= - 0.43 p= < 0.01  r= - 0.50 p= < 0.01 | Not Identified    Health Belief Model  (comparative risk)  Ajzen (1991) Extension of TPB |
| **Hand Hygiene**  O’Boyle *et al.* (2001)  Jenner *et al.* (2002) | Critical Care Nurses  Nurses, Therapists and Health Care Assistants | Intensity of Activity  Personal Responsibility  Time Availability  Number and Location of sinks | NR    r= 0.42 p= < 0.01 | r = - 0.32 p < 0.05 (Observed)  r= 0.36 p= < 0.01  r=0.40 p= < 0.01  r=0.30 p= < 0.01 | Not Identified  Health Belief Model |
| **General Guideline Use**  Maue and Segal *et al.* (2004) | Physicians and Advanced Registered Nurse Practitioners | Internal barriers  External Barriers  Perceived Barriers | r = - 0.50, P <0.0029  r = - 0.39, P <0.025  NR | r= - 0.47 =<0.006 | NR |
| **Specialist Guideline Use**  Bolman *et al.* (2002)  (Smoking Cessation)  Beatty and Beatty (2004) (Safety Guidelines)  Bonetti *et al.* (2010)  (Placing Fissure Sealants)  Leitlen *et al.* (2011)  (Smoking Cessation)  Buenestado *et al.* (2013) | Nurses    Anaesthetists    Dental Practitioners  Nurse Practitioners  Practice Nurses  Physicians | Perceived Simplicity  Level of experience  Nurses own smoking behaviour  Personal Norm (Habit) (no pre-op visit)  Personal Norm (Habit) (no cockpit checks)  Personal Norm (Habit) (silence alarms)  Habit    Satisfaction  Facilitating Factors (educational preparation)  Habit | r = 0.65 p < 0.01  r = 0.26 p < 0.05  r= 0.22 p < 0.05  r=0.62 p=< 0 .01  r=0.79 p=< 0 .01  r=0.47 p=< 0.01  Beta 0.59 p=< 0.001  Odds ratio 0.42  R= 0.98 p=<.05  Mean difference p=<.05 | NR    NR          Beta 0.35 p=< 0.001    NR  NR | NR  Ajzen (1991) Extension of TPB    Blackman (1974) Operant Learning Theory  Extension of Technology Acceptance Model (TAM) |
